# Prenatal substance exposure and infant neurodevelopment: a review of magnetic resonance imaging studies

**DOI:** 10.3389/fnhum.2025.1613084

**Published:** 2025-08-20

**Authors:** Leela Shah, Christy D. Yoon, Alessandra M. LaJeunesse, Lilly G. Schirmer, Emma W. Rapallini, Elizabeth M. Planalp, Douglas C. Dean

**Affiliations:** ^1^Department of Neuroscience Training Program, University of Wisconsin-Madison, Madison, WI, United States; ^2^Waisman Center, University of Wisconsin-Madison, Madison, WI, United States; ^3^Department of Pediatrics, University of Wisconsin-Madison, Madison, WI, United States; ^4^Department of Medical Physics, University of Wisconsin-Madison, Madison, WI, United States

**Keywords:** prenatal substance exposure, magnetic resonance imaging, infant brain development, diffusion MRI, structural MRI, functional MRI, developmental outcomes

## Abstract

Amid the ongoing global substance use crisis, prenatal health research has increasingly focused on the impact of both licit and illicit substance use on fetal development, and in particular brain development. Magnetic resonance imaging (MRI) has become a critical non-invasive tool for investigating how such exposures influence the developing brain. In this review, we summarize findings from 25 peer-reviewed studies that leverage structural, functional, and diffusion MRI to examine the effects of prenatal exposure to alcohol, opioids, methamphetamines, cocaine, nicotine, or cannabis. Particular attention was given to studies that paired infant MRI data with developmental outcomes. Existing research has implicated cortical and sub-cortical gray and white matter regions across substance exposures, with associations between MRI findings and developmental outcomes in infancy. We identify key limitations in the existing literature, including small sample sizes, lack of control for prematurity, sex, co-occurring exposures, limited developmental assessment, and insufficient longitudinal follow-up. We highlight the need for future research linking early neuroimaging findings to developmental outcomes, particularly in large, diverse, and nationally representative cohorts. Such work is essential for informing evidence-based policies, clinical guidelines, and targeted interventions for families impacted by prenatal substance exposure.

## Introduction

There is rising concern surrounding substance use (defined here as use of licit or illicit substances of abuse), with prenatal substance exposures posing particular public health concern (Narkowicz et al., 2013). For example, in the United States, alcohol consumption among pregnant individuals has been reported by the Centers for Disease Control at 13.5%, with a 5.2% rate of binge drinking (Gosdin, 2022). While tobacco use during pregnancy is declining, additional substances of abuse remain a concern due to the opioid crisis and rising rates of cannabis use. In a 2020 U.S. survey, 8% of respondents reported cannabis use, 8% reported nicotine use, 0.4% reported opioid use and 0.3% reported cocaine use during pregnancy, with other stimulant use varying across reports (SAMHSA, 2020). The prevalence of methamphetamine in pregnancy in the U.S. is thought to be close to 0.19% (Young-Wolff et al., 2022). Worldwide, the prevalence of substance use in pregnancy varies significantly. Specific to alcohol, the prevalence of alcohol use in pregnancy was recently reported to range from 0 to 0.5% in northern Africa and the Middle East to greater than 40% in Russia, Denmark, Belarus, and Ireland (Popova et al., 2017). Prenatal substance use prevalence varies across nations likely due to a number of factors, including cultural norms, reporting practices, healthcare access, and stigma (Chiandetti et al., 2017). However, the widespread use of substances prenatally underscores the critical relevance of studying the effects of prenatal substance exposures (Tavella et al., 2020), which may pose concern for both short-term and long-term child development.

Prenatal substance exposure poses significant risks to fetal development. Different substances may exert unique effects on the developing fetus, but a shared concern is their potential to disrupt critical processes in brain development that can have enduring consequences. Prenatal substance use is associated with clinically recognized specific effects; for instance, fetal alcohol spectrum disorder (FASD) (Riley et al., 2011) may be seen in infants with alcohol exposure and neonatal opioid withdrawal syndrome (NOWS) is associated with prenatal opioid exposure (Conradt et al., 2019). However, long-term sequelae are also associated with prenatal substance exposures, specifically exposure to alcohol, nicotine or tobacco, cannabis, or methamphetamine, include developmental delays, deficits in cognition and attention, issues with impaired visuospatial working memory, and mental health challenges, which have been reported throughout childhood and adolescence (De Genna et al., 2022; Ernst et al., 2001; Harst et al., 2021; Lambert and Bauer, 2012; Ross et al., 2015; Townsel et al., 2021). The reported long-term neurodevelopmental sequelae of substance exposures reflects vulnerability in the developing brain to substances of abuse. Because each substance interacts differently with fetal biology (Bailey and Diaz-Barbosa, 2018; Ortigosa et al., 2012), outcomes may depend on several factors, including dosage, timing of exposure, and the presence of other environmental or substance-related exposures (Ross et al., 2015). Understanding how substance use exposure potentially disrupts neurodevelopment requires a close examination of the complex biological processes that occur in utero. Specifically, identifying the mechanisms through which substances alter fetal brain development can help explain the wide variability in outcomes in affected infants and guide strategies for early detection and intervention.

### Impact of substance exposures on fetal neurodevelopment

The time from gestation through the first year of life is a critical period during which the brain matures most rapidly (Tierney and Nelson, 2009), and insults during this critical period have been associated with enduring developmental consequences (Andersen, 2003; Matthews et al., 2018; Raschle et al., 2012). Subcortical neurons begin forming as early as 10 weeks of gestation, and white matter myelination, axonal synaptogenesis, dendritic arborization, and formation of functional connections occur during the second and third trimesters (Adams-Chapman, 2009; Dufford et al., 2021; Ouyang et al., 2019; Stiles and Jernigan, 2010). The timing of substance exposure may thus yield different neurodevelopmental signatures, with additional consequences if the exposure is associated with preterm birth (Adams-Chapman, 2009).

Various neurotransmitter systems play fundamental roles in guiding neuronal proliferation, migration, synaptogenesis, and circuit refinement during critical periods of fetal brain development. During gestation, substances may cross the placenta through diffusion and transport mechanisms, affecting key processes and leading to alterations in these neurotransmitter systems, potentially altering neural organogenesis, growth, and/or function, depending on target receptors (Eléfant et al., 2020). Disruptions to these systems can therefore have cascading effects on neural architecture. Specifically, in the developing fetus, substance exposures may be associated with decreased dopamine synthesis and release (cocaine, methamphetamine, and opioids, Boggess and Risher, 2022; Little et al., 2021; Tomášková et al., 2020),a compensatory up-regulation of dopamine D1 and D2 receptor density (cocaine and opioids, Boggess and Risher, 2022; Little et al., 2021), altered dopamine release and serotonin turnover (nicotine, Little et al., 2021), and disrupted glutamatergic and GABAergic neuron development and signaling (alcohol, cannabis, and nicotine, Little et al., 2021). For example, GABA and glutamate not only act as neurotransmitters but also as trophic factors that regulate early neuronal differentiation and cortical patterning (Egbenya et al., 2021; Ji et al., 2024). Substance exposures are also linked to changes in glucocorticoid receptor expression, inflammatory cytokine production, and HPA axis functioning (Frank et al., 2011; Franks et al., 2019; Salisbury et al., 2009). Additionally, substance exposure may be linked to physiological alterations within the pregnant individual, including alterations to placental vasculature and physiology (Ortigosa et al., 2012), that impact the delivery of oxygen and nutrients to the fetus and further affect neurodevelopment (Rees and Harding, 2004). This brief overview necessarily simplifies a highly complex and dynamic set of neurodevelopmental processes, yet highlights the potential mechanisms through which prenatal substance impacts the developing fetus. The neurobiology of prenatal substance exposure is further described in Ortigosa et al. (2012) and Ross et al. (2015).

Understanding the shared and unique neurochemical pathways of substances of abuse is essential to interpreting their effects on the developing brain. While each substance of abuse exhibits distinct pharmacologic profiles, there are shared mechanisms between neurochemical pathways (US Department of Health and Human Services, 2016). Depressants, such as alcohol, interact with neurotransmitter systems in the brain, including GABA, glutamate, and other systems to produce a depressant effect (Heilig and Egli, 2006). Opioids bind to opioid receptors in the brain, leading to dopamine release from the nucleus accumbens (Wang, 2019). Relatedly, stimulants including cocaine and methamphetamine increase the amount of dopamine and norepinephrine in the brain’s reward circuitry (Koob, 1992). Although nicotine acts on nicotinic acetylcholine receptors and cannabis interacts with cannabinoid receptors, these substances also share the ultimate effect of activating the dopamine system throughout the brain (Chayasirisobhon, 2021; Miller and Picciotto, 2016). While the targets of substances of abuse differ, altered neurotransmitter function within reward circuitry across substance exposures may contribute to overlapping substance exposure profiles.

The differential neurodevelopmental effects of prenatal substance exposure have been studied using cellular and animal models, which have detailed the pharmacologic actions, neurotransmission effects, implicated regions, and behavioral effects of various substance exposures (Eiden et al., 2023). Animal studies have reported concentration-dependent effects of substance exposures, regions impacted across substance exposures (including the basal ganglia and reward network) and concentration-dependent cytotoxicity in offspring exposed to substances prenatally (Eiden et al., 2023; Kuhn et al., 2019; Lovinger and Alvarez, 2017; Ross et al., 2015). This foundational work provides potential biological mechanisms to explain observed relations between prenatal substance exposure and neurodevelopmental outcomes in humans, using mechanistic experimental models that are not feasible in humans (Ross et al., 2015). To translate findings from animal models to clinical populations, magnetic resonance imaging (MRI) may be used as a non-invasive, high-resolution method for studying early human brain development (Ouyang et al., 2019).

### MRI studies of prenatal substance exposure

Although several reviews have examined the use of MRI to study individuals exposed to substances in utero (Donald et al., 2015a; Irner, 2012; Sanjari Moghaddam et al., 2021), these often focus on brain changes observed later in childhood and adolescence, overlooking the earliest manifestations of brain disruption following prenatal substance exposure. Two reviews have focused specifically on the neonatal and infant periods, when the brain is rapidly developing and may be particularly vulnerable to disruption (Dufford et al., 2021; Pulli et al., 2018). Another extended the scope to include functional neuroimaging and electroencephalography studies from infancy to early adulthood, with an emphasis on non-alcohol substance exposure (Morie et al., 2019). These works synthesize structural and functional MRI findings in infants with a wide range of prenatal exposures, including alcohol, nicotine, illicit substances, pharmaceuticals, maternal obesity, and inflammatory conditions. While they highlight alterations in brain volume, microstructure, and functional connectivity, they offer limited insight into how these changes relate to early developmental outcomes.

Building upon these prior reviews, we provide a targeted overview of the effects of prenatal substance exposure on brain development using infant MRI research, focusing on studies performed during the neonatal and infant periods and examining how neuroimaging findings relate to immediate developmental outcomes a dimension that has received limited attention in prior work. Unlike previous reviews, which either emphasize older developmental stages (Donald et al., 2015a; Sanjari Moghaddam et al., 2021) or include a broader selection of neuroimaging modalities (e.g., EEG and fNIRS in Morie et al., 2019), our review is the first to examine findings from structural, functional, and diffusion MRI modalities specifically in infants during the earliest postnatal stages. We excluded other functional modalities such as EEG and fNIRS to maintain a consistent focus on MRI-based methodologies, which provide both high spatial resolution and multi-modal anatomical and functional insights into early brain development (Dubois et al., 2021). In narrowing our scope, we bridge a critical gap in the literature and offer a foundation for identifying neurobiological markers that could inform intervention efforts in the earliest postnatal stages.

## Methods

### Search strategy

The literature search for this review occurred on October 12th, 2024, covering papers that were published between January 1st, 2000 and October 12th, 2024. The following inclusion criteria were used to select studies: (a) be empirical and published in a scholarly, peer-reviewed journal in English; (b) include human infants from gestation to 1 year old; (c) include a group exposed to one or more of the 6 most commonly used substances of abuse during gestation (SAMHSA, 2020), including alcohol, nicotine, opioids, cocaine, methamphetamine, or cannabis; and (d) utilize an MRI modality. Treatment-related studies were included if they met inclusion criteria a-d and reported relationships between substance exposure and brain MRI findings in alignment with our goal of summarizing MRI findings associated with prenatal substance exposure. While we were interested in reported relationships between MRI signatures and developmental outcomes, developmental assessment was not an inclusion criterion as we were interested in substance use’s effects on the brain directly as well as immediate developmental outcomes. Exclusion criteria included case studies, review articles, non-English articles, articles that did not meet all inclusion criteria (age, prenatal substance exposure, and MRI), and non-human studies.

The literature search was conducted across Academic Search Premier, ERIC, MedLine, PsycArticles, PsycINFO, and PubMed by the first author (LS), with the initial search We searched using the following keywords: (“neonat*” OR “*newborn*” OR “*infant*” OR “prem*” OR “bab*”) AND (“*substance*” OR “*drug*” OR “*alcohol*”) AND (“*MRI*” OR “*magnetic resonance imaging*” OR “*magnetic*” OR “*resonance*” OR “*imaging*”). Filters were applied to limit results to peer-reviewed, empirical studies published in English, excluding case studies and review articles. The initial search yielded a total of 3,544 articles.

### Study selection

Following duplicate removal, the titles and abstracts of the 3,356 articles were screened for eligibility by the first author (LS), leading to the removal of 3,330 articles based on the inclusion and exclusion criteria. The remaining 26 manuscripts were assessed by one of EGR or LGS, with additional review by LS, with 4 additional articles excluded upon in-depth review. The manuscript references of the remaining 22 articles were reviewed by EGR, LGS, and/or LS, and 3 additional articles were identified for inclusion based on bibliography review. The search process led to 25 articles deemed appropriate for inclusion in this review. During manuscript preparation, all included articles were approved by EWR, LGS, and LS, with no discrepancies in article inclusion decisions between authors.

### Data extraction

Relevant information for each study included the primary substance exposure of interest, sample sizes of the exposed and control groups, sample age range, MRI techniques and parameters, MRI regions of interest (ROIs), developmental measures, and the main reported outcomes.

## Results

This review includes 25 studies published between 2009 and 2023 that investigated prenatal exposure to alcohol (*n* = 7), opioids (*n* = 7), methamphetamines (*n* = 4), cocaine (*n* = 2), nicotine (*n* = 2), cannabis (*n* = 1), or polysubstance exposure (*n* = 2). While some studies included non-focal substance exposures as controls, only those examining the unique effects of multiple substances were included in the polysubstance exposure category. The imaging modalities employed were structural MRI (T1-, T2-, and proton density-weighted; *n* = 9); resting-state functional MRI (rsfMRI; *n* = 9); and diffusion tensor imaging (DTI), including probabilistic tractography, tract-based spatial statistics (TBSS), and region-of-interest methods (*n* = 7). See Tables 1–7 for details from each of the 25 reviewed studies.

**Table 1:** Alcohol exposure: summary of main findings of the included studies (*n* = 7), including MRI and developmental outcomes.

Reference and Cohort	Sample n exposed/control	Sample age at imaging	MRI technique and parameters	Brain regions	Developmental outcome and age at testing	Main reported outcomes
Alcohol						
Donald et al. (2015c) Drakenstein Child Health Study, Univ. of Cape Town	28/28 Excluded participants with positive urine screening at 28–32 weeks gestation for non-alcohol drugs of abuse Alcohol use quantified as at least 2 times weekly or 2 or more drinks per occasion in at least 1 trimester	2–4 weeks postnatal (infants born < 36 weeks gestation excluded)	DTI (whole brain TBSS and regions of interest) FA, MD, AD, RD	Association fibers, brainstem tracts, projection fibers, commissural fibers	Dubowitz Behavior and Abnormal Signs Subscale Age: 2–4 weeks postnatal	Alcohol-exposed infants showed lower AD in the right superior longitudinal fasciculus. Lower FA in the right inferior cerebellar peduncle in exposed infants was positively associated with behavioral subscale scores and increased MD in the right inferior cerebellar peduncle was negatively associated with behavioral subscale scores.
Donald et al. (2016) Drakenstein Child Health Study, Univ. of Cape Town	13/14 Excluded participants with positive urine screening at 28–32 weeks gestation for non-alcohol drugs of abuse Alcohol use quantified as at least 2 times weekly or 2 or more drinks per occasion in at least 1 trimester	2–4 weeks postnatal (infants born < 36 weeks gestation excluded)	Resting-state functional connectivity Seed-based functional connectivity	Sensorimotor intrinsic functional connectivity networks	Dubowitz Behavior and Abnormal Signs Subscales Age: 2–4 weeks postnatal	Alcohol exposure was associated with higher connectivity between somatosensory, motor, brainstem/thalamic, and striatal intrinsic networks. Exposed and control groups showed no differences in the Dubowitz Subscales.
Donald et al. (2015b) Drakenstein Child Health Study, Univ. of Cape Town	28/45 Excluded participants with positive urine screening at 28–32 weeks gestation for non-alcohol drugs of abuse Alcohol use quantified as at least 2 times weekly or 2 or more drinks per occasion in at least 1 trimester	2–4 weeks postnatal (infants born < 36 weeks gestation excluded)	T2-weighted gray matter volumes Regional volumes	90 gray matter regions of interest	Dubowitz Behavior and Abnormal Signs Subscales, Bayley Scales of Infant and Toddler Development (BSID) Age: 2–4 weeks postnatal (Dubowitz), 6 months (BSID)	Alcohol exposure was associated with smaller overall gray matter volume and smaller left hippocampal, bilateral amygdala, and left thalamic volumes in exposed infants. Exposed infants with larger regional volumes in the temporal and frontal lobes had higher scores on both developmental scales.
Jacobson et al. (2017) Drakenstein Child health Study, Univ. of Cape Town	32/11 Participants divided into heavy/binge-drinking (14 or more drinks/weeks or 4 or more drinks per occasion) and little-to-no exposure (not meeting heavy/binge-drinking criteria)	6–40 days postnatal, corrected for GA if born < 37 weeks GA	T1-weighted and Proton-density brain volumes Regional brain volume	Corpus callosum	N/A	Alcohol-exposed neonates had smaller corpus callosum volumes. Controlled for prenatal exposure to smoking, cannabis, and methamphetamine.
Roos et al. (2021) Drakenstein Child Health Study, Univ. of Cape Town	11/14 Alcohol use quantified as more than 1 standard drink per week or 2 + binge drinking episodes (4 + drinks per occasion) during pregnancy	2–4 weeks postnatal (infants born < 36 weeks gestation excluded)	Resting-state fMRI Functional connectivity	Global functional hub arrangement and regional connectivity across the whole brain	N/A	Exposed neonates had temporal and limbic hubs within global functional networks, while control infants had more distributed networks. Regional networks of exposed neonates showed predominant connectivity in subcortical and occipital regions, while networks of control neonates showed predominant connectivity in parietal and occipital regions.
Taylor et al. (2015) Drakenstein Child Health Study, Univ. of Cape Town	11/20 Excluded participants with methamphetamine or cocaine use. Minimal cannabis use was allowed. Alcohol use quantified as 4 or more drinks on at least one occasion or 14 + drinks/week.	38–44 weeks GA* *One infant scanned prior to 38 weeks	DTI probabilistic tractography	Transcallosal pathways, cortico-spinal projection fibers, and cortico-cortical association fibers	N/A	Alcohol exposure was associated with lower AD and MD. All white matter tracts analyzed, with the strongest associations in the medial and inferior white matter. Controlled for tobacco smoking.
Warton et al. (2021) Drakenstein Child Health study, Univ. of Cape Town	50*/0 *N = 27 infants of birthing parents randomized to receive high-dose choline supplementation Alcohol use quantified as at least 2 standard drinks / day or two or more binge drinking episodes.	1–7 weeks postnatal (infants excluded if < 32 weeks GA)	Structural MRI with multi-echo FLASH sequence Regional volumes	Caudate nuclei, putamen, hippocampus, cerebellar hemispheres, cerebellar vermis, corpus callosum	Fagan Test of Infant Intelligence Age: 6.5 months, 12 months	Choline-treated exposed infants showed larger bilateral thalamic, bilateral caudate, right putamen, and corpus callosal volumes. Higher maternal choline adherence was positively associated with these brain volumes. Larger right putamen and corpus callosal volumes in choline-supplemented infants were associated with higher recognition memory at 12 months. Controlled for cannabis and tobacco use.

The Dubowitz is a validated scale for the motor and behavioral states in newborns, which includes an ‘abnormal signs’ cluster that evaluates for posture, tremor, and startle (Dubowitz et al., 2005). Fagan Test of Infant Intelligence is a narrow-band test validated to identify impairments in attention, reaction time, recognition memory, and processing speed associated with alcohol exposed infants (Fagan, 2012). AD, axial diffusivity; FA, fractional anisotropy; MD, mean diffusivity; RD, radial diffusivity; TBSS, Tract-based Spatial Statistics. N/A = not applicable.

**Table 2:** Opioid exposure: summary of main findings of the included studies (*n* = 7), including MRI and developmental outcomes.

Reference and cohort	Sample n exposed/control	Sample age at imaging	MRI technique and parameters	Brain regions	Developmental outcome and age at Testing	Main reported outcomes
Opioids (any or multiple)						
Jiang et al. (2022) Univ. of North Carolina at Chapel Hill	21/28 Polysubstance exposure not excluded Opioid exposure quantified by maternal history and/or urine toxicology at time of delivered and confirmed with neonatal toxicology	6 weeks postnatal (infants born < 37 weeks excluded)	Resting-state fMRI Inter-network and intra-network functional connectivity	Fronto-parietal, ventral attention, default mode, dorsal attention, sensorimotor, visual, and limbic edge-centric matrices	N/A	Inter-network connectivity was further disrupted than intra-network connectivity within the visual, subcortical, and default mode networks in exposed infants. The greatest differentiation between control and exposed infants was seen in the amygdala, nucleus accumbens, and inferior temporal gyrus.
Liu et al. (2022) cedars-Sinai medical center	81*/28 * *N* = 31 exposed to methadone or buprenorphine * *N* = 53 exposed to opioids (not methadone or buprenorphine) * *N* = 39 with non-opioid exposure, including cocaine, nicotine, alcohol, cannabis, stimulants, depressants, or other substances Polysubstance exposure not excluded Opioid exposure quantified by maternal history and/or urine toxicology at time of delivered and confirmed with neonatal toxicology	2 weeks postnatal (no correction for prematurity)	Resting-state functional connectivity Heatmap whole-brain functional connectivity analysis	Functional connectivity throughout the brain	N/A	Infants exposed to methadone or buprenorphine had significantly fewer opioid-exposure-related alterations in limbic and frontal connections, compared to infants exposed to opioids (not methadone or buprenorphine). Infants receiving buprenorphine/methadone treatment had residual alterations in some limbic and subcortical connections relative to non-exposed controls.
Merhar et al. (2021) Cincinnati children’s hospital	29/42 Polysubstance exposure not excluded Opioid exposure quantified as at least 4 weeks of exposure to any opioid	40–48 weeks postmenstrual age (infants born < 37 weeks excluded)	T2-weighted brain volumes Regional volumes	58 bilateral white and gray matter regions	N/A	Exposed infants had smaller relative volumes of deep gray matter, bilateral thalamic ventrolateral nuclei, bilateral insular white matter, bilateral subthalamic nuclei, brainstem, and cerebrospinal fluid. Exposed infants had larger relative volumes of the right cingulate gyrus white matter and left occipital lobe white matter.
Radhakrishnan et al. (2021) Indiana Univ.	10*/12 *9 participants were undergoing medication assisted treatment (7 buprenorphine, 2 methadone) Polysubstance exposure not excluded Opioid exposure quantified using medical records and self-report questionnaire	<48 weeks corrected GA (infants born < 37 weeks excluded)	Resting-state fMRI Seed-based connectivity	Right/left amygdala, cortical regions, and precuneus	N/A	Opioid-exposed infants had higher connectivity between the bilateral amygdalae and the medial prefrontal cortex, precuneus, and other cortical subregions compared to the control infants, with asymmetries between left and right amygdala connectivity. There were no significant correlations between morphine milligram equivalent dose and connectivity.
Yuan et al. (2014) Brains, opioids, and babies collaborative group, new south wales	16/0 *N = 14 exposed to methadone, 4 to buprenorphine, and 11 used more than one opioid. Polysubstance exposure not excluded Opioid exposure quantified using self-report. Exposure confirmed with neonatal urine and meconium testing.	Mean age 1.5 weeks postnatal (infants born < 37 weeks excluded)	T1-weighted gray and white matter volumes Regional volumes	Subcortical regions, cerebellum, cortical gray and white matter	N/A	Exposed infants had significantly smaller basal ganglia than the population mean volume and significantly larger lateral ventricles than the population mean volume.
Opioids (methadone)						
Monnelly et al. (2018) Univ. of Edinburgh	20/20 Polysubstance exposure not excluded Methadone exposure quantified as prescription for methadone for opioid use disorder	37–42 weeks postmenstrual age (infants born < 37 weeks excluded)	DTI with tract-based spatial statistics FA, RD, MD, AD	Global white matter skeleton	N/A	Exposed infants had higher median white matter FA and decreased FA in the centrum semiovale, inferior longitudinal fasciculus, and external capsules. Exposed infants had higher RD in the internal capsule and inferior longitudinal fasciculus.
Walhovd et al. (2012) Canterbury Methadone in pregnancy study, Univ. of Oslo, Norway	13/7 Polysubstance exposure not excluded Excluded infants with fetal alcohol syndrome or heavy alcohol usage reported in pregnancy. Opioid exposure quantified as enrollment in methadone therapy for opioid use disorder in the third trimester of pregnancy.	13–44 days postnatal (36–42 weeks GA, no correction for prematurity)	DTI with probabilistic tractography MD	Superior longitudinal fasciculus, inferior longitudinal fasciculus and white matter skeleton	N/A	Higher MD in the inferior longitudinal fasciculus and superior longitudinal fasciculus of exposed infants was found in both the voxelwise and tract-based analyses.

Study cohorts are listed to illustrate cohort overlap across studies. Gestational age (GA) is included where reported. AD, axial diffusivity; FA, fractional anisotropy; MD, mean diffusivity; RD, radial diffusivity. N/A = not applicable.

**Table 3:** Methamphetamine exposure: summary of main findings of the included studies (*n* = 4), including MRI and developmental outcomes.

Reference and cohort	Sample n exposed/control	Sample age at imaging	MRI technique and parameters	Brain regions	Developmental outcome and age at testing	Main reported outcomes
Methamphetamine						
Chang et al. (2016) Univ. of Hawaii at Manoa	68*/71 *N = 36 exposed to methamphetamine/ tobacco, 32 exposed to tobacco Excluded participants with alcohol use > 3 drinks per month during pregnancy, polysubstance use, or cocaine use Methamphetamine exposure quantified by self-report	1–3 sessions between 0–4 months postnatal (no correction for prematurity)	DTI FA, MD, AD, RD	Corpus callosum, caudate, corona radiata, internal capsule, globus pallidus, putamen, thalamus, corticospinal tract	Amiel-Tison Neurological Assessment at Term Age: newborn, 3–4 months	Methamphetamine/ tobacco- and tobacco-exposed females had lower FA in the anterior corona radiata than control females. Methamphetamine/ tobacco-exposed males had smaller FA and larger diffusivities in the superior and posterior corona radiata, with normalization by 3 months. Tobacco-exposed infants showed persistently lower axial diffusion in the thalamus and internal capsule. Methamphetamine/ tobacco-exposed infants showed delayed developmental trajectories on active muscle tone and total neurologic scores, which normalized by 3–4 months.
Warton et al. (2018a) Drakenstein Child Health Study, Univ. of cape town	18/21 Polysubstance exposure not excluded Methamphetamine exposure quantified as 2 + uses per month	1–4 weeks postnatal* *Infants born prior to 34 weeks scanned at 7–9 weeks postnatal	T1-weighted brain volumes Regional volumes	Caudate, putamen, thalamus, hippocampus, vermis, cerebellum	N/A	Exposed infants had reduced bilateral caudate and thalamic volumes. Analyses controlled for cannabis and tobacco use.
Warton et al. (2018b) Drakenstein Child Health Study, Univ. of Cape Town	11/12 Polysubstance exposure not excluded Methamphetamine exposure quantified as 2 + uses per month	1–5 weeks postnatal (no correction for prematurity)	DTI Probabilistic tractography FA, AD, RD	OFC, caudate, nucleus accumbens, putamen, hippocampus, midbrain, amygdala	N/A	Higher exposure was associated with lower FA in connections between the striatum and midbrain, orbitofrontal cortex, and associated limbic structures. Analyses controlled for cannabis and tobacco use.
Warton et al. (2020) Drakenstein Health Study, Univ. of Cape Town	11/12 Polysubstance exposure not excluded Methamphetamine exposure quantified as 2 + uses per month	1–5 weeks postnatal* *1 premature infant born at 31 weeks GA scanned at 9 weeks postnatal	DTI Probabilistic tractography FA, AD, RD	Association fibers, projection fibers, commissural fibers	N/A	Higher exposure was associated with lower FA in bilateral association and projection and commissural networks (corticospinal tracts, internal capsule, optic radiations, thalamic radiations, uncinate, occipitofrontal fasciculus, superior and inferior longitudinal fasciculi). Higher exposure was associated with lower AD in the right association and increased RD in the right projection and bilateral association networks. Analyses controlled for cannabis and tobacco use.

Study cohorts are listed to illustrate cohort overlap across studies. Gestational age (GA) is included where reported. The Amiel-Tison Neurological Assessment at Term is a method to determine risk of neurologic injury in neonates (Gosselin et al., 2005). AD, axial diffusivity; FA, fractional anisotropy; MD, mean diffusivity; RD, radial diffusivity. N/A = not applicable.

**Table 4:** Cocaine exposure: summary of main findings of the included studies (*n* = 2), including MRI and developmental outcomes.

Reference and cohort	Sample n exposed/control	Sample age at imaging	MRI technique and parameters	Brain regions	Developmental outcome and age at testing	Main reported outcomes
Cocaine						
Grewen et al. (2014) Univ. of North Carolina at Chapel Hill	73*/46 *N = 33 exposed to cocaine with or without additional substances *N = 40 exposed to nicotine, alcohol, opiates, and/or selective serotonin reuptake inhibitors without cocaine exposure Substance exposure quantified by self-report	Mean age 5 weeks postnatal (infants born < 36 weeks excluded)	Dual contrast MRI (T1w, T2w) Regional volumes	Cortical regions: right/left dorsal, ventral, prefrontal, frontal, parietal, occipital	N/A	Infants with prenatal cocaine exposure had lower total gray matter and regional gray volume in prefrontal and frontal regions relative to controls and infants with non-cocaine drug exposure. Infants with prenatal cocaine exposure had greater total CSF volume and regional CSF volumes in prefrontal, frontal, and parietal relative to controls and infants with non-cocaine drug exposure. Analyses controlled for non-cocaine substance exposures.
Salzwedel et al. (2016) Univ. of north Carolina at chapel hill	88*/64 * *N* = 45 exposed to cocaine with or without other exposures. *N = 43 others exposed to non-cocaine substances. Substance exposure quantified by self-report	2–6 weeks postnatal (infants born < 32 weeks or > 42 weeks excluded)	Resting-state fMRI Functional connectivity	Thalamus and cortical regions	Bayley Scales of Infant and Toddler Development Age: 3 months	Cocaine-exposed infants exhibited hyper-connectivity between the thalamus and frontal regions and hypo-connectivity between the thalamus and motor-related regions. Thalamo-frontal connectivity in cocaine exposed infants was related to lower cognitive and fine motor skills and thalamo-motor connectivity showed a positive relationship with composite motor skills. Cocaine by selective-serotonin-reuptake-inhibitor interactions were detected.

Study cohorts are listed to illustrate cohort overlap across studies. Gestational age (GA) is included where reported. The Bayley Scales of Infant and Toddler Development is a validated scoring system to assess cognitive, language, and motor development (Bayley, 2005). N/A = not applicable.

**Table 5:** Nicotine exposure: summary of main findings of the included studies (*n* = 2), including MRI and developmental outcomes.

Reference and cohort	Sample n exposed / control	Sample age at imaging	MRI technique & parameters	Brain regions	Developmental outcome and age at testing	Main reported outcomes
Nicotine (tobacco smoking)						
Ekblad et al. (2010) IMAC-MIND Consortium, Germany	43/190 Alcohol exposure not excluded Nicotine exposure quantified by self-report	Imaging at term of infants born <32 weeks gestation or <37 weeks gestation with birth weight <1,500 g	T1-weighted gray matter volumes Regional volumes	Cerebral, cerebellar, frontal lobe, medulla, pons, basal ganglia, and thalami	N/A	Exposed infants had smaller frontal and cerebellar volumes than control infants. Analyses controlled for alcohol use.
MahabeE-Gittens et al. (2023) Cincinnati infant neurodevelopment early prediction study, Univ. of Cincinnati	50/345 Polysubstance exposure not excluded Nicotine exposure quantified by self-report	Imaging at 39–44 weeks post- conception of infants born at <32 weeks gestation	Regional volumes, global brain abnormality score, global efficiency of structural connectome	White matter, cortical gray matter, deep gray matter, cerebellum	N/A	Exposed infants had higher median Global Brain Abnormality Score and diffuse white matter abnormality volume than control infants. Exposed infants also had lower global efficiency and total brain tissue volume. Preterm birth mediated 0–29% of the effect of prenatal tobacco smoke exposure on brain abnormality outcomes. Analyses controlled for opioid and cannabis use.

Study cohorts are listed to illustrate cohort overlap across studies. Gestational age (GA) is included where reported. The Global Brain Abnormality Score accounts for white matter, cortical gray matter, deep gray matter, and cerebellar regions based on an MRI scoring system (Kidokoro et al., 2013). NA = not applicable.

**Table 6:** Cannabis exposure: summary of main findings of the included studies (n = 1), including MRI and developmental outcomes.

Reference and cohort	Sample n exposed/control	Sample age at imaging	MRI technique and parameters	Brain regions	Developmental outcome and age at testing	Main reported outcomes
Cannabis						
Grewen et al. (2015) Univ. of North Caroline at Chapel Hill	43*/23 *N = 20 exposed to cannabis with or without additional substances *N = 23 exposed to nicotine, alcohol, opiates, and/or selective serotonin reuptake inhibitors without cannabis exposure Cannabis exposure quantified using self-report	2–6 weeks postnatal (infants born < 36 weeks excluded)	Resting-state functional connectivity Seed-based functional connectivity analysis	Subcortical seed regions with high fetal CB1R expression (amygdala, hippocampus, putamen, anterior/posterior insula, caudate, and anterior/posterior thalamus)	N/A	Cannabis-exposed infants demonstrated hypoconnectivity in the insula, anterior insula-cerebellum, right caudata-cerebellum, right caudate-right fusiform gyrus/inferior occipital, left caudate-cerebellum circuits. Both exposed groups (cannabis exposed and non-cannabis exposed) had hyper-connectivity of the left amygdala seed with the orbital frontal cortex and hypo-connectivity of the posterior thalamus seed with the hippocampus.

Study cohorts are listed to illustrate cohort overlap across studies. Gestational age (GA) is included where reported. N/A = not applicable.

**Table 7:** Polysubstance exposure: summary of main findings of the included studies (*n* = 2), including MRI and developmental outcomes.

Reference and cohort	Sample n exposed / control	Sample age at imaging	MRI technique and parameters	Brain regions	Developmental outcome and age at testing	Main reported outcomes
Polysubstance exposure						
Salzwedel et al. (2015) Univ. of North Carolina at Chapel Hill	73*/46 *N = 33 exposed to cocaine with or without additional substances *N = 40 exposed to nicotine, alcohol, opiates, and/or selective serotonin reuptake inhibitors without cocaine exposure Substance exposure quantified by self-report	Within ~4 weeks postnatal	Resting-state functional connectivity Seed-based whole-brain functional connectivity analysis	Functional connectivity between seed regions (amygdala and insula) and frontal and sensorimotor cortices	N/A	Exposed infants had connectivity disruptions in the amygdala-frontal, insula-frontal, and insula-sensorimotor circuits. A cocaine-specific effect was seen within a subregion of the amygdala-frontal network.
Salzwedel et al. (2020) Univ. of North Carolina at Chapel Hill	75/58 *N = 75 exposed to cocaine, cannabis, alcohol, nicotine, selective serotonin reuptake inhibitors, and/or opioids Cannabis exposure quantified by self-report	2–6 weeks postnatal (no correction for prematurity)	Resting-state functional connectivity Intersubject variability in functional connectivity between seed regions	Whole-brain analysis with 222 seed regions	Bayley Scales of Infant and Toddler Development Age: 3 months	~5% of whole-brain functional connections were affected by substance exposure, particularly within higher-order brain networks. Substance-specific effects included associations between nicotine exposure and bilateral medial and right lateral prefrontal regions, cocaine exposure and bilateral cingulate and left middle frontal areas, opioid exposure and right angular and left middle frontal gyrus connectivity. Regions showing significant drug effects were significantly correlated with poorer behavioral outcome measures, with a mediation role of the brain functional connectivity between exposure status and cognitive/language outcomes.

Study cohorts are listed to illustrate cohort overlap across studies. Gestational age (GA) is included where reported. The Bayley Scales of Infant and Toddler Development is a validated scoring system to assess cognitive, language, and motor development (Bayley, 2005). N/A = not applicable.

### Alcohol

The 7 studies that investigated prenatal alcohol exposure are listed in Table 1. The number of exposed infants from each study ranged from 11 to 50, all assessed within the first 7 weeks of life. All data were collected in Cape Town, South Africa as part of a large child health study. Although cohort overlap could not be precisely determined, substantial duplication across studies is likely. Collectively, data on alcohol use during pregnancy was associated with disrupted white matter maturation in cortical regions such as the superior longitudinal fasciculus and cerebellar regions (Donald et al., 2024; Taylor et al., 2015), reduced subcortical, corpus callosum, and basal ganglia volumes (Donald et al., 2015b; Jacobson et al., 2017; Warton et al., 2021), and elevated resting state functional connectivity (rsFC) in somatosensory, motor, occipital, brainstem, and subcortical networks in alcohol-exposed infants in the first weeks of life (Donald et al., 2016; Roos et al., 2021). Brain structure and microstructure changes were associated with abnormal neonatal behaviors at birth (Donald et al., 2015c) and were linked to lower intelligence at 12 months (Warton et al., 2021), with some evidence suggesting that choline supplementation may partially mitigate these effects (Warton et al., 2021).

### Opioids

Seven studies examined prenatal opioid exposure (see Table 2), including a total of 174 exposed infants across separate cohorts. Two of these studies reported brain imaging findings in methadone-exposed infants. All data was collected prior to 9 weeks of life. None of the included studies examined behavioral outcomes associated with brain imaging findings. Overall, these findings suggest that opioid exposure is associated with heterogenous white matter alterations across cortical regions (Monnelly et al., 2018; Walhovd et al., 2012), disrupted subcortical volumes (Merhar et al., 2021; Yuan et al., 2014), and abnormal functional connectivity in reward-related brain networks (Jiang et al., 2022; Liu et al., 2022; Radhakrishnan et al., 2021), with possible attenuation in the context of medication for opioid use disorder (MOUD) therapy (Liu et al., 2022). Importantly, no literature to our knowledge has compared infant neuroimaging findings in opioid exposure with developmental assessments.

### Methamphetamine

Four studies examined the effects of prenatal methamphetamine exposure on early brain development, with study cohorts of methamphetamine exposed infants ranging from 11–36 infants (see Table 3). Three studies included overlapping cohorts. Initial imaging was conducted within the first 9 weeks of life, with some longitudinal data extending to 16 weeks of life (Chang et al., 2016). Studies included both term (>37 weeks GA) and preterm (<37 weeks GA) infants. One study compared methamphetamine exposure (with or without tobacco exposure) to tobacco exposure; this study was included in the methamphetamine category rather than the polysubstance category given the focus on methamphetamine over and above the use of tobacco, rather than on tobacco alone. Overall, prenatal methamphetamine exposure is associated with smaller subcortical volumes in the basal ganglia and hippocampus (Warton et al., 2018a) and disrupted white matter microstructure in commissural, association, and projection regions (Chang et al., 2016; Warton et al., 2020; Warton et al., 2018b). Exposure was associated with delayed active muscle tone development at birth which normalized in early infancy (Chang et al., 2016). One longitudinal study reported sex-specific findings, including persistently disrupted microstructure in the anterior corona radiata in methamphetamine exposed females, and disrupted microstructure in the superior and posterior corona radiata in methamphetamine exposed males that normalized by 3 months (Chang et al., 2016).

### Cocaine

Two studies within the same cohort ranging from 73 to 88 exposed infants (with likely overlap between study cohorts) examined relationships between prenatal cocaine exposure and brain development in infants less than 6 weeks of age (see Table 4). Together, these findings suggest that prenatal cocaine exposure is associated with both decreases in cortical gray matter volumes (Grewen et al., 2014) and altered functional connectivity between the thalamus and cortical regions, with early evidence of cognitive and motor developmental delays in exposed infants (Salzwedel et al., 2016). Altered thalamo-cortical connectivity was associated with lower cognitive, fine motor, and composite motor scores at 3 months in exposed infants, suggesting a relationship between neonatal functional connectivity and development in exposed infants (Salzwedel et al., 2016).

### Nicotine

Two studies examined the effects of prenatal nicotine exposure on brain development in a total of 93 preterm infants, all born before 32 weeks GA. No included studies assessed behavioral outcomes in relation to imaging findings (see Table 5). Overall, these studies indicate that prenatal nicotine exposure is linked to smaller frontal and cerebellar brain volumes (Ekblad et al., 2010) and diffuse white matter abnormalities (Mahabee-Gittens et al., 2023) in preterm infants. However, there is a lack of data on term-born infants, non-tobacco nicotine exposures, and associations with developmental outcomes.

### Cannabis

Only 1 study reported associations between cannabis exposure and brain development in a sample of 43 exposed term (> 36 weeks GA) infants (see Table 6). This work revealed cannabis-associated reductions in caudate and insular connectivity with the cerebellum, occipital regions, and fusiform regions in exposed infants (Grewen et al., 2015). No work to our knowledge has examined relationships between brain imaging findings, such as those reported in Grewen et al. (2015), and developmental outcomes in infancy.

### Polysubstance exposure

Two studies within the same cohort of term and preterm infants examined the effects of polysubstance exposure on infant brain development, with study cohorts ranging from 73–75 exposed infants with likely overlap between study samples (see Table 7). Together, these findings suggest that polysubstance exposure during gestation contributes to widespread disruptions in functional connectivity (Salzwedel et al., 2020; Salzwedel et al., 2015) and may increase risk for early developmental delays, particularly in language and motor domains (Salzwedel et al., 2020).

## Discussion

### Overview of findings

Current evidence highlights substance-specific differences in early brain development between infants with and without prenatal substance exposure. This review examined neuroimaging differences associated with prenatal exposure and explored how these imaging signatures relate to developmental outcomes, where such associations were reported. Across studies, we identified substance-related associations with brain structure and function, including volumetric alterations, microstructural differences, and variations in rsfMRI. These findings spanned cortical and subcortical gray and white matter regions, including fronto-limbic and reward networks, motor regions including the cerebellum and brainstem, hippocampus, thalamus, basal ganglia, and white matter relay tracts.

### MRI signatures of prenatal substance exposure

**Figure 1:**
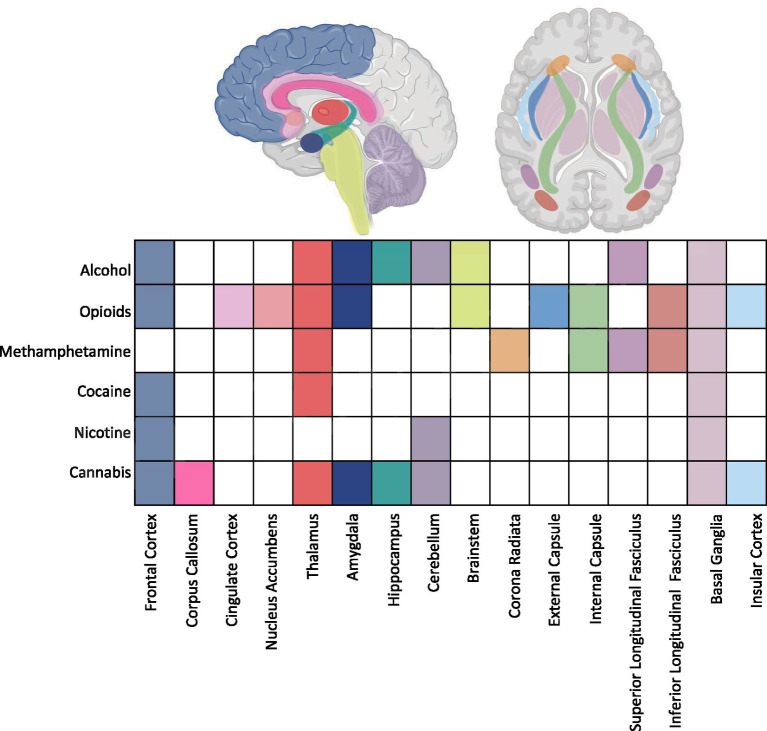
Summary of imaging findings across substance exposures highlighting target regions. Aspects of this image created in BioRender. Shah, L. (2025) https://BioRender.com/tukao8z.

Research points to differences between substance-exposed infants and non-exposed infants in regional volumes measured using structural MRI, microstructural markers of brain maturity as measured by diffusion MRI techniques, and regional and global connectivity patterns studied using rsfMRI techniques. Figure 1 summarizes regions implicated by neuroimaging data across substance exposures.

#### Structural MRI

During early development, both white and gray matter regions exhibit remarkable growth, resulting in volume expansion throughout the brain (Groeschel et al., 2010). Structural imaging findings among included studies consistently indicate that infants with prenatal substance exposure tend to have smaller gray and white matter volumes and larger cerebrospinal fluid (CSF) volumes across various brain regions. The basal ganglia, thalamus, hippocampus, amygdala, and cortex are particularly affected. Notably, volume normalization in regions such as the thalamus, caudate, putamen, and corpus callosum was observed in alcohol-exposed infants treated postnatally with choline, suggesting potential for early intervention (Warton et al., 2021). Infancy represents a critical period of rapid brain growth, particularly in white and gray matter, and slower growth during this stage has been associated with poorer developmental outcomes, suggesting that the observed structural differences may have long-term implications for substance-exposed infants (Lind et al., 2011; Peterson et al., 2003). Most studies utilized regional and global volumetric quantification approaches. However, none employed advanced quantitative MRI techniques, such as relaxometry, magnetization transfer imaging, magnetic resonance spectroscopy, or quantitative susceptibility mapping. These techniques are capable of capturing subtle developmental differences and are highly sensitive to tissue composition, including water content, myelination, and iron (Nossin-Manor et al., 2013; Sled and Nossin-Manor, 2013). The absence of these modalities limits our ability to characterize the biological underpinnings of the observed volumetric changes and offers opportunities for future studies.

#### rsfMRI

During infancy, brain circuits exhibit differing patterns of functional connectivity changes that generally follow a pattern of higher overall functional connectivity, higher within-network connectivity, and few aberrant connections between distinct networks (Hoff et al., 2013). The resting-state fMRI studies included in this review suggest that prenatal substance exposure to alcohol, opioids, cocaine, and cannabis is associated with widespread alterations in functional connectivity, particularly in cortical–subcortical networks. Findings support a pattern of altered development of functional connectivity in exposed infants, with aberrantly strong subcortical–cortical connections and less coherence within sensorimotor (alcohol and cocaine, Donald et al., 2016; Salzwedel et al., 2016), limbic (alcohol, opioids, cocaine, and cannabis, Grewen et al., 2015; Jiang et al., 2022; Liu et al., 2022; Radhakrishnan et al., 2021; Roos et al., 2021; Salzwedel et al., 2016) and cerebellar (cannabis, Grewen et al., 2015) networks. Interestingly, fronto-limbic changes appear attenuated in infants exposed to MOUD compared to those exposed to non-MOUD opioids, although disruptions in certain limbic-subcortical pathways persist (Liu et al., 2022). Similar patterns of altered functional connectivity have been observed in older children and adolescents with prenatal substance exposure, where they have been linked to poorer executive function and general developmental outcomes (Sirnes et al., 2018; Smith et al., 2016; Vishnubhotla et al., 2024). These early life connectivity disruptions may reflect neural circuit alterations that contribute to later behavioral and cognitive challenges seen in this population, but longitudinal research is needed to determine how early connectivity differences evolve over time and whether they can reliably predict later outcomes.

#### DTI

It is well established that increasing FA and decreasing diffusivity reflect key microstructural processes occurring during early brain development, such as increased axonal density, more complex fiber organization, and ongoing myelination (Friedrich et al., 2020; Song et al., 2002). DTI parameters are therefore considered sensitive markers of tissue microstructure and have been linked to favorable developmental outcomes (Cancelliere et al., 2013; Lebel and Deoni, 2018; Qiu et al., 2008). DTI findings reviewed here suggest that prenatal substance exposure is associated with broad alterations of brain microstructure and affect a range of developing white matter tracts, including the corpus callosum, corona radiata, superior and inferior longitudinal fasciculi, and internal and external capsules. Lower FA in major white matter tracts may represent less myelination and/or reduced organization (Lebel and Deoni, 2018). Moreover, studies reporting higher RD and lower AD across white matter tract supports the hypothesis of reduced myelination and altered fiber organization in substance-exposed infants (Harsan et al., 2006; Song et al., 2002). Patterns of white matter MD differences between exposed and control groups were less consistent across studies, with most studies reporting increased MD and (Taylor et al., 2015) reporting decreased MD in white matter tracts in exposed infants. This variability may reflect regional and substance-specific differences in the impact of prenatal substance exposure across the brain, where effects do not necessarily occur uniformly or in the same direction across all white matter regions. Additionally, since RD and AD both contribute to the calculation of MD, different relationships between prenatal substance exposures and RD or AD may also contribute to the opposite directionality of the effect on MD across substances and regions (Counsell et al., 2006; Lebel et al., 2019; Winklewski et al., 2018). Further, existing literature has reported patterns of more developed white matter (higher FA and lower MD) of fronto-limbic regions in socioeconomically disadvantaged infants (Lean et al., 2022), suggesting an adaptation to environmental stress. The higher maturity observed in fronto-limbic regions has not been documented in substance exposure, but it may explain the deviations from the generally observed decrease in white matter maturity in substance-exposed infants. Importantly, the diffusion imaging studies reviewed here relied solely on conventional DTI modeling. More advanced techniques such as diffusion kurtosis imaging (DKI) and neurite orientation dispersion and density imaging (NODDI), among others, may offer additional sensitivity and specificity to underlying neurobiological alterations (DiPiero et al., 2023; Steven et al., 2014; Zhang et al., 2012). However, these approaches are limited by longer acquisition times and higher b-values, which may reduce feasibility in neonatal populations (DiPiero et al., 2023).

The neuroimaging associations observed in substance-exposed infants are likely driven by multiple complex and interacting biological mechanisms. Prenatal substance use may disrupt maternal systemic and placental physiology, potentially limiting the availability of critical nutrients required for healthy brain development (Ross et al., 2015). These disruptions may underlie the reductions in gray and white matter volumes, compromised microstructural integrity, and altered resting-state functional connectivity observed in exposed infants (Eléfant et al., 2020; Rees and Harding, 2004). Additionally, prior research in adolescents has identified structural and functional brain differences, such as increased neural activation, reduced brain volumes, and diminished white matter integrity in regions involved in reward processing and reinforcement (De Genna et al., 2022; Ernst et al., 2001; Sirnes et al., 2018; Vishnubhotla et al., 2024). These neurodevelopmental changes are associated with a heightened risk for substance use behaviors; therefore, similar early-life imaging signatures in substance-exposed infants represent neurobiological vulnerabilities that may predispose individuals to substance use disorders later in life (Abu and Roy, 2021; Nygaard et al., 2017; Oei, 2018; Squeglia and Gray, 2016).

### Brain regions and circuits of interest

Evidence from the included studies suggests both overlapping and distinct patterns of brain alterations associated with prenatal exposure to different substances. Several regions appear consistently vulnerable across substances, while others show exposure-specific associations (Figure 1).

Across substances, including opioids, alcohol, methamphetamine, cannabis, and polysubstance use, exposed infants consistently exhibit alterations in structures within the reward and limbic systems, including the amygdala, nucleus accumbens, hippocampus, basal ganglia, and insula that are associated with these exposures. Given the widespread effects of prenatal substance exposures on these regions, reward and limbic differences may represent a shared neural phenotype of prenatal substance exposure. The involvement of these networks in infants exposed to substances parallels alterations within the reward system reported among adolescents with substance use disorders (Salmanzadeh et al., 2020; Squeglia and Gray, 2016). Early disruptions in these circuits may compromise affective processing and reward sensitivity, possibly mediated by perturbations in dopamine signaling due to substance exposure (Sesack and Grace, 2010). Ultimately, reward circuitry changes evident in infancy could contribute to heightened vulnerability for later-life substance use (Dodge et al., 2019), although this risk likely emerges from a complex interplay of genetic predisposition, neurobiological consequences of exposure, and broader environmental and social determinants (Abu and Roy, 2021; Nygaard et al., 2017; Oei, 2018).

Further, differences in primary sensory and motor relay centers, such as the thalamus, internal and external capsules, and corticospinal tract, were reported in infants with prenatal exposure to opioids, alcohol, and methamphetamine. These findings are consistent with adult studies linking substance use with alterations in similar relay structures (Huang et al., 2018; Pando-Naude et al., 2021) and suggest a vulnerability of sensorimotor pathways, potentially driven by disruptions in excitatory and inhibitory (glutamate and GABAergic) signaling which are affected across substance exposures and are critical to early thalamocortical and corticospinal development (Luján et al., 2005; Nishimaru and Kakizaki, 2009; Amitai, 2001). Given the role of these tracts in motor coordination and sensorimotor integration, disruptions may underlie the motor impairments observed in substance-exposed infants and children (Salzwedel et al., 2016; Willford et al., 2010).

Additionally, cortical disruptions, particularly within the frontal and prefrontal cortex were observed in infants exposed to opioids, cocaine, cannabis, nicotine, or polysubstance use (Ekblad et al., 2010; Grewen et al., 2014, 2015; Radhakrishnan et al., 2021; Salzwedel et al., 2020; Salzwedel et al., 2015, 2016). These regions, particularly within the prefrontal cortex, have frequently been implicated in adults and adolescents with substance use disorders and have been associated with cognitive and executive function impairments (Goldstein and Volkow, 2011; Squeglia et al., 2009). Dysfunction in these regions may contribute to the cognitive findings observed in infancy (Donald et al., 2015b; Salzwedel et al., 2020; Salzwedel et al., 2016). This may be driven by disruptions to synaptic pruning, myelination, or neurochemical systems such as dopamine and GABA, which shape early cortical development (Ojeda and Ávila, 2019).

Substance-specific patterns also emerged. For example, cerebellar differences were most consistently reported in infants exposed to alcohol, nicotine, and cannabis, suggesting selective vulnerability of cerebellar networks to these substances. This substance-specific pattern may be related to the high concentration of GABA (target for alcohol), cholinergic and nicotinic (targets for nicotine), and cannabinoid (target for cannabis) receptors in the cerebellum (Hauser et al., 2003; Hsiao et al., 1999; Takahashi and Linden, 2000), although future work in large samples is needed to further assess the effects of additional substance exposures on cerebellar development. Given the cerebellum’s role in sensorimotor coordination and emerging links to cognitive function, its alteration may underlie both motor and attentional deficits observed in exposed populations (Konczak and Timmann, 2007; Lyu et al., 2025).

While not the primary focus of this review, regions implicated in human infant neuroimaging studies are well-supported by preclinical literature on prenatal substance exposure (Ross et al., 2015). Animal studies have found that prenatal stimulants may affect dopaminergic system development (Thompson et al., 2009), while prenatal cocaine exposure may alter basal ganglia, hippocampus, amygdala, and cortical development (Dow-Edwards et al., 1990). Alcohol exposure in utero has been linked to abnormalities in the neocortex, hippocampus, cerebellum, and other regions (Guerri, 2002). Additionally, murine models have implicated the hippocampus and basal ganglia (opioids), hippocampus and cortex (nicotine), and the cerebellum, thalamus, hypothalamus, and hippocampus (cannabis) in substance-related developmental disruption (Benevenuto et al., 2022; Byrnes and Vassoler, 2018; Roy et al., 2002). This overlap between human neuroimaging and preclinical data provides a valuable foundation for future mechanistic studies.

In sum, the current literature suggests a core set of regions, including the limbic and reward systems, white matter relay tracts, and the frontal cortex, that are commonly affected across exposure types, possibly reflecting shared neurodevelopmental mechanisms (e.g., dysregulation of dopamine, glutamate, and GABA systems). At the same time, certain regions such as the cerebellum appear to show more substance-specific vulnerability, underscoring the importance of both common and unique pathways through which prenatal exposures impact early brain development. Nonetheless, results are preliminary, within small samples with significant cohort overlap between studies, so additional substance-specific effects and effects across substance exposures may be revealed with future work. Implicated regions aligned with both adolescent studies and animal models of prenatal substance exposure. Further whole-brain analyses examining effects of prenatal substance exposure may reveal additional relationships across the brain.

### Developmental consequences of prenatal substance exposure

Prenatal substance exposure has been linked to a range of developmental outcomes in infancy, but the existing neuroimaging literature provides limited insight into the neurobiological mechanisms of these associations. Across alcohol and methamphetamine exposures, neuroimaging findings were associated with impairments in central and peripheral neurological function in the neonatal period. In substance exposed infants, neuroimaging differences relative to healthy controls were associated with poorer cognitive and fine motor skills (cocaine exposure) and worse cognitive and language skills (polysubstance exposure) on the BSID-III at 3 months of age. These associations provide early insight into how substance-related alterations in brain structure and function may translate into long-term developmental challenges. Nonetheless, while a number of included studies reported abnormal developmental findings at birth in substance exposed infants, one study reported normalization of these findings by 3–4 months of age, highlighting the need for longitudinal follow-up of developmental findings (Chang et al., 2016). Moreover, by design, few included studies evaluated development beyond the neonatal period, making it challenging to contextualize the long-term functional relevance of neuroimaging findings.

Thus, it remains unclear whether these outcomes reflect a delay in developmental progression, permanent deficits in specific neural systems, or a combination of both mechanisms. Such ambiguity underscores the need for longitudinal studies to track developmental trajectories over time in exposed infants. Importantly, protective factors, including supportive caregiving, parental mental health, and enriched home environments, have been shown to moderate adverse outcomes in adolescence and may serve as critical buffers against early brain vulnerability (Bada et al., 2012; Motz et al., 2011). Future neuroimaging studies should examine how these protective factors influence the relationship between prenatal substance exposure and brain development.

### Clinical intervention studies

While not the main focus of this work, two studies explored the impact of medications for substance use. One study found normalized regional brain volumes in the thalamus, basal ganglia, and corpus callosum in pregnant people with alcohol exposure treated with high-dose choline supplementation during pregnancy, which related to better recognition memory at 12 months, compared with non-treated alcohol exposed infants (Warton et al., 2021). Another study found a reduction in opioid-associated changes in rsFC in infants exposed to methadone therapy relative to infants exposed to opioids without treatment (Liu et al., 2022; Radhakrishnan et al., 2021). These studies provide promising evidence for treatment-related effects on infant brain development, albeit in small sample sizes. Future work should continue to evaluate treatment-related effects of medications for substance use in pregnancy on infant brain and developmental outcomes.

### Limitations in the current literature

One overarching limitation is the small sample sizes across studies, with exposed infant cohorts ranging from 10 to 88 participants. Additionally, most studies excluded occasional users but did not consistently address critical variables such as dosage or timing of substance exposure which are likely to influence outcomes and clinical translation of findings. A tightly orchestrated sequence of neurodevelopmental events unfolds throughout gestation, with varied developmental trajectories across brain circuits and regions, so the timing of substance exposures across trimesters may have varying impacts on brain development and merits further examination with sufficiently powered analyses (Andescavage et al., 2017; Xu et al., 2022). Only 2 studies within the same cohort (Warton et al., 2020; Warton et al., 2018b) examined dose effects, and none evaluated timing of exposure. Polysubstance use presents an additional methodological challenge. Although some studies focused on or included data from polysubstance-exposed groups, most did not control for the influence of multiple exposures. Therefore, observed neural differences may reflect synergistic effects or confounding interactions between commonly co-ingested substances such as alcohol, nicotine, and cannabis. Furthermore, although some studies linked neuroimaging signatures to developmental outcomes, these associations were typically based on cross-sectional assessments conducted at the time of imaging. Few studies explored infant developmental outcomes beyond the first weeks of life. No included studies assessed whether imaging findings were associated with clinical diagnoses, such as FASD or NOWS, which could help contextualize these neuroimaging findings. Similarly, no studies included comparisons between neuroimaging findings and physiological measures such as inflammatory markers or hormonal changes, which are known to be disrupted in fetal substance exposure (Frank et al., 2011; Franks et al., 2019; Salisbury et al., 2009).

Further, the existing body of literature is limited in its control of confounding factors and co-occurring influences that could contribute to observed differences between substance-exposed infants and healthy control infants. These factors include maternal psychosocial health, use of prescription medications, physical health status, and socioeconomic conditions, which may independently impact fetal brain development and interact with substance exposure (Mravčík et al., 2020). Although studies variably excluded or controlled for preterm birth, prematurity itself is linked to altered neurodevelopment and is more common among substance-exposed neonates (Bada et al., 2005; Ortinau and Neil, 2015; Umer et al., 2023). One study found that prematurity mediated nearly one-third of the effect of tobacco exposure on brain development, highlighting its significant influence (Mahabee-Gittens et al., 2023).

Sex differences also remain underexplored. Only one study in this review reported sex-specific findings (Chang et al., 2016), despite established sex-related differences in brain development and susceptibility to prenatal insults, including substance exposure (Dipietro and Voegtline, 2015; Hines, 2010; Kaczkurkin et al., 2019; Terasaki et al., 2016). Future studies should prioritize evaluating sex-specific effects to more precisely identify vulnerable subgroups.

Finally, the predominantly cross-sectional nature of included studies limits the ability to determine whether observed neural differences represent long-term changes or delays that might resolve with early intervention. Longitudinal imaging studies will be crucial to understanding whether and how brain alterations evolve across development. While infant neuroimaging findings mirror those reported in older children and adolescents, including disruptions in cortical, cerebellar, and fronto-limbic structures, as well as in the basal ganglia, corpus callosum, amygdala, and hippocampus, postnatal experiences and social environments continue to shape neural development over time (Derauf et al., 2009; Willford et al., 2016). These dynamic interactions emphasize the need for long-term follow-up studies.

### Current review: methodological limitations

Certain limitations of this review should be noted. First, despite efforts to develop a comprehensive search strategy, including reviewing the bibliographies of the studies in this review, it is possible that some relevant studies may not have been included due to the limitations of the selected databases and search terms. Variability in how studies are indexed and the terminologies used by the authors may have contributed to the omission of pertinent literature. Additionally, to include all existing literature on imaging findings in infants with substance exposure, diffusion-weighted, structural, and functional MRI are included in this review, making direct comparisons between these studies challenging. Moreover, since the initial database search was conducted by a single author, this may introduce a limitation regarding potential selection bias.

Within modalities, different statistical approaches and techniques for delineating regions of interest were applied. For example, among the DTI analyses, tract-based spatial statistics were leveraged in two studies; four studies utilized probabilistic tractography to delineate regions of interest and one study used atlas-segmented regions of interest. Similarly, brain volumes could not be directly compared across the existing structural data, which applied T1−/T2-weighted MRI, T1-weighted MRI, T2-weighted MRI, or proton density-weighted MRI to estimate brain volumes. These methodological inconsistencies may account for some differences observed across studies.

Additionally, several studies originated from the same research groups or used overlapping samples, potentially biasing findings. Some infants were scanned beyond the neonatal window, introducing postnatal environmental influences that could confound results. Furthermore, the focus on infancy may limit the generalizability of findings to later developmental stages, although existing reviews in older populations complement this work (Irner, 2012; Sanjari Moghaddam et al., 2021).

### Future directions

Despite these limitations, the findings of this review point to meaningful differences in brain development associated with prenatal substance exposure, particularly in the context of polysubstance use, which warrants further study given its high prevalence (Jarlenski and Krans, 2021). Future research should continue to evaluate the distinct and combined effects of individual substances on brain development while accounting for moderating variables such as sex, gestational age, birth weight, maternal mental health, and socioeconomic status (Dufford et al., 2021; Pulli et al., 2018).

Additional investigation into affected neural circuits, especially those involved in reward processing, sensory-motor integration, cognition, and white matter connectivity, is warranted. Researchers should leverage modern diffusion-weighted models and quantitative structural techniques to further investigate affected brain regions. Longitudinal studies are especially needed to clarify how early neural alterations relate to evolving developmental trajectories and functional outcomes.

Further, examining the impact of the timing and dosage of prenatal substance exposure will be critical for disentangling its specific neurodevelopmental effects. Understanding whether pharmacological or psychosocial interventions can mitigate adverse outcomes is essential to inform clinical care and public health policy. Large, diverse, and nationally representative samples will be necessary to contextualize substance-related effects within broader social and environmental risk factors.

## Conclusion

Evidence from structural, functional, and diffusion MRI data suggests that prenatal substance exposure is associated with measurable alterations in infant brain development. These effects vary by substance type, co-exposures, and affected brain regions, and are associated with early developmental outcomes. However, small sample sizes and imprecise exposure measurement limit the strength of current conclusions. Failure to account for additional sources of disadvantage, sex, and prematurity also limits current work. Continued large-scale, longitudinal research is needed to refine our understanding of how prenatal exposures impact brain development, inform clinical practice, and guide supportive policies for affected families.

## References

[ref1] Abu Y. Roy S. (2021). Prenatal opioid exposure and vulnerability to future substance use disorders in offspring. Exp. Neurol. 339:113621. doi: 10.1016/j.expneurol.2021.113621, PMID: 33516730 PMC8012222

[ref2] Adams-Chapman I. (2009). Insults to the developing brain and impact on neurodevelopmental outcome. J. Commun. Disord. 42, 256–262. doi: 10.1016/j.jcomdis.2009.03.010, PMID: 19423130

[ref3] Amitai Y. (2001). Thalamocortical synaptic connections: efficacy, modulation, inhibition and plasticity. Rev. Neurosci. 12, 159–174. doi: 10.1515/REVNEURO.2001.12.2.159, PMID: 11392456

[ref4] Andersen S. L. (2003). Trajectories of brain development: point of vulnerability or window of opportunity? Neurosci. Biobehav. Rev. 27, 3–18. doi: 10.1016/S0149-7634(03)00005-8, PMID: 12732219

[ref5] Andescavage N. N. du Plessis A. McCarter R. Serag A. Evangelou I. Vezina G.. (2017). Complex trajectories of brain development in the healthy human fetus. Cereb. Cortex 27, 5274–5283. doi: 10.1093/cercor/bhw306, PMID: 27799276 PMC6074870

[ref6] Bada H. S. Bann C. M. Whitaker T. M. Bauer C. R. Shankaran S. LaGasse L.. (2012). Protective factors can mitigate behavior problems after prenatal cocaine and other drug exposures. Pediatrics 130, e1479–e1488. doi: 10.1542/peds.2011-3306, PMID: 23184114 PMC3507246

[ref7] Bada H. S. Das A. Bauer C. R. Shankaran S. Lester B. M. Gard C. C.. (2005). Low birth weight and preterm births: etiologic fraction attributable to prenatal drug exposure. J. Perinatol. 25, 631–637. doi: 10.1038/sj.jp.7211378, PMID: 16107872

[ref8] Bailey N. A. Diaz-Barbosa M. (2018). Effect of maternal substance abuse on the fetus, neonate, and child. Pediatr. Rev. 39, 550–559. doi: 10.1542/pir.2017-0201, PMID: 30385584

[ref9] Bayley N. (2005). Bayley Scales of Infant and Toddler Development, Third Edition (Bayley--III®) [Database record]. APA PsycTests.

[ref10] Benevenuto S. G. M. Domenico M. D. Yariwake V. Y. Dias C. T. Mendes-da-Silva C. Alves N. d. O.. (2022). Prenatal exposure to *Cannabis* smoke induces early and lasting damage to the brain. Neurochem. Int. 160:105406. doi: 10.1016/j.neuint.2022.105406 35970295

[ref11] Boggess T. Risher W. C. (2022). Clinical and basic research investigations into the long-term effects of prenatal opioid exposure on brain development. J. Neurosci. Res. 100, 396–409. doi: 10.1002/jnr.24642, PMID: 32459039

[ref12] Byrnes E. M. Vassoler F. M. (2018). Modeling prenatal opioid exposure in animals: current findings and future directions. Front. Neuroendocrinol. 51, 1–13. doi: 10.1016/j.yfrne.2017.09.001, PMID: 28965857 PMC5649358

[ref13] Cancelliere A. Mangano F. T. Air E. L. Jones B. V. Altaye M. Rajagopal A.. (2013). DTI values in key white matter tracts from infancy through adolescence. AJNR Am. J. Neuroradiol. 34, 1443–1449. doi: 10.3174/ajnr.A3350, PMID: 23370472 PMC8051496

[ref14] Chang L. Oishi K. Skranes J. Buchthal S. Cunningham E. Yamakawa R.. (2016). Sex-specific alterations of white matter developmental trajectories in infants with prenatal exposure to methamphetamine and tobacco. JAMA Psychiatry 73, 1217–1227. doi: 10.1001/jamapsychiatry.2016.2794, PMID: 27829078 PMC6467201

[ref15] Chayasirisobhon S. (2021). Mechanisms of action and pharmacokinetics of Cannabis. Perm. J. 25, 1–3. doi: 10.7812/TPP/19.200, PMID: 33635755 PMC8803256

[ref16] Chiandetti A. Hernandez G. Mercadal-Hally M. Alvarez A. Andreu-Fernandez V. Navarro-Tapia E.. (2017). Prevalence of prenatal exposure to substances of abuse: questionnaire versus biomarkers. Reprod. Health 14:137. doi: 10.1186/s12978-017-0385-3, PMID: 29070078 PMC5657059

[ref17] Conradt E. Flannery T. Aschner J. L. Annett R. D. Croen L. A. Duarte C. S.. (2019). Prenatal opioid exposure: neurodevelopmental consequences and future research priorities. Pediatrics 144:e20190128. doi: 10.1542/peds.2019-0128, PMID: 31462446 PMC6759228

[ref18] Counsell S. J. Shen Y. Boardman J. P. Larkman D. J. Kapellou O. Ward P.. (2006). Axial and radial diffusivity in preterm infants who have diffuse white matter changes on magnetic resonance imaging at term-equivalent age. Pediatrics 117, 376–386. doi: 10.1542/peds.2005-0820, PMID: 16452356

[ref19] De Genna N. M. Willford J. A. Richardson G. A. (2022). Long-term effects of prenatal cannabis exposure: pathways to adolescent and adult outcomes. Pharmacol. Biochem. Behav. 214:173358. doi: 10.1016/j.pbb.2022.173358, PMID: 35216971 PMC8911923

[ref20] Derauf C. Kekatpure M. Neyzi N. Lester B. Kosofsky B. (2009). Neuroimaging of children following prenatal drug exposure. Semin. Cell Dev. Biol. 20, 441–454. doi: 10.1016/j.semcdb.2009.03.001, PMID: 19560049 PMC2704485

[ref21] DiPiero M. Rodrigues P. G. Gromala A. Dean D. C. (2023). Applications of advanced diffusion MRI in early brain development: a comprehensive review. Brain Struct. Funct. 228, 367–392. doi: 10.1007/s00429-022-02605-8, PMID: 36585970 PMC9974794

[ref22] Dipietro J. A. Voegtline K. M. (2015). The gestational foundation of sex differences in development and vulnerability. Neuroscience 342:4. doi: 10.1016/j.neuroscience.2015.07.068 26232714 PMC4732938

[ref23] Dodge N. C. Jacobson J. L. Jacobson S. W. (2019). Effects of fetal substance exposure on offspring substance use. Pediatr. Clin. N. Am. 66, 1149–1161. doi: 10.1016/j.pcl.2019.08.010, PMID: 31679604 PMC6986376

[ref24] Donald K. A. Eastman E. Howells F. M. Adnams C. Riley E. P. Woods R. P.. (2015a). Neuroimaging effects of prenatal alcohol exposure on the developing human brain: a magnetic resonance imaging review. Acta Neuropsychiatr. 27, 251–269. doi: 10.1017/neu.2015.12, PMID: 25780875

[ref25] Donald K. A. Fouche J. P. Roos A. Koen N. Howells F. M. Riley E. P.. (2015b). Alcohol exposure in utero is associated with decreased gray matter volume in neonates. Metab. Brain Dis. 31, 81–91. doi: 10.1007/s11011-015-9771-0, PMID: 26616173 PMC6556617

[ref26] Donald K. A. Hendrikse C. J. Roos A. Wedderburn C. J. Subramoney S. Ringshaw J. E.. (2024). Prenatal alcohol exposure and white matter microstructural changes across the first 6-7 years of life: a longitudinal diffusion tensor imaging study of a south African birth cohort. NeuroImage Clinic. 41:103572. doi: 10.1016/j.nicl.2024.103572, PMID: 38309186 PMC10847766

[ref27] Donald K. A. Ipser J. C. Howells F. M. Roos A. Fouche J.-P. Riley E. P.. (2016). Interhemispheric functional brain connectivity in neonates with prenatal alcohol exposure: preliminary findings. Alcohol. Clin. Exp. Res. 40, 113–121. doi: 10.1111/acer.12930, PMID: 26727529 PMC6556616

[ref28] Donald K. A. Roos A. Fouche J.-P. Koen N. Howells F. M. Woods R. P.. (2015c). A study of the effects of prenatal alcohol exposure on white matter microstructural integrity at birth. Acta Neuropsychiatr. 27, 197–205. doi: 10.1017/neu.2015.35, PMID: 26022619 PMC6465963

[ref29] Dow-Edwards D. L. Freed L. A. Fico T. A. (1990). Structural and functional effects of prenatal cocaine exposure in adult rat brain. Dev. Brain Res. 57, 263–268. doi: 10.1016/0165-3806(90)90052-Z, PMID: 2073724

[ref30] Dubois J. Alison M. Counsell S. J. Hertz-Pannier L. Hüppi P. S. Benders M. J. N. L. (2021). MRI of the neonatal brain: a review of methodological challenges and neuroscientific advances. J. Magn. Reson. Imaging 53, 1318–1343. doi: 10.1002/jmri.27192, PMID: 32420684 PMC8247362

[ref31] Dubowitz L. Ricciw D. Mercuri E. (2005). The Dubowitz neurological examination of the full-term newborn. Ment. Retard. Dev. Disabil. Res. Rev. 11, 52–60. doi: 10.1002/mrdd.20048, PMID: 15856443

[ref32] Dufford A. J. Spann M. Scheinost D. (2021). How prenatal exposures shape the infant brain: insights from infant neuroimaging studies. Neurosci. Biobehav. Rev. 131, 47–58. doi: 10.1016/j.neubiorev.2021.09.017, PMID: 34536461

[ref33] Egbenya D. L. Aidoo E. Kyei G. (2021). Glutamate receptors in brain development. Childs Nerv. Syst. 37, 2753–2758. doi: 10.1007/s00381-021-05266-w, PMID: 34164719

[ref34] Eiden R. D. Perry K. J. Ivanova M. Y. Marcus R. C. (2023). Prenatal substance exposure. Annu. Rev. Dev. Psychol. 5 (Volume 5, 2023), 19–44. doi: 10.1146/annurev-devpsych-120621-043414, PMID: 40874035 PMC12380384

[ref35] Ekblad M. Korkeila J. Parkkola R. Lapinleimu H. Haataja L. Lehtonen L. (2010). Maternal smoking during pregnancy and regional brain volumes in preterm infants. J. Pediatr. 156, 185–190.e1. doi: 10.1016/j.jpeds.2009.07.061 19818449

[ref36] Eléfant E. Hanin C. Cohen D. (2020). “Chapter 26—pregnant women, prescription, and fetal risk” in Handbook of clinical neurology. eds. Gallagher A. Bulteau C. Cohen D. Michaud J. L., vol. 173. (Amsterdam, Netherlands: Elsevier), 377–389. 10.1016/B978-0-444-64150-2.00027-7 32958185

[ref37] Ernst M. Moolchan E. T. Robinson M. L. (2001). Behavioral and neural consequences of prenatal exposure to nicotine. J. Am. Acad. Child Adolesc. Psychiatry 40, 630–641. doi: 10.1097/00004583-200106000-00007, PMID: 11392340

[ref38] Fagan J. F. (2012). Fagan Test of Infant Intelligence (FTII) [Database record]. APA PsycTests. doi: 10.1037/t04887-000

[ref39] Frank M. G. Watkins L. R. Maier S. F. (2011). Stress- and glucocorticoid-induced priming of neuroinflammatory responses: potential mechanisms of stress-induced vulnerability to drugs of abuse. Brain Behav. Immun. 25, S21–S28. doi: 10.1016/j.bbi.2011.01.005, PMID: 21256955 PMC5654377

[ref40] Franks A. L. Berry K. J. DeFranco D. B. (2019). Prenatal drug exposures and neurodevelopmental programming of glucocorticoid signaling. J. Neuroendocrinol. 32:e12786. doi: 10.1111/jne.12786 31469457 PMC6982551

[ref41] Friedrich P. Fraenz C. Schlüter C. Ocklenburg S. Mädler B. Güntürkün O.. (2020). The relationship between axon density, myelination, and fractional anisotropy in the human Corpus callosum. Cereb. Cortex 30, 2042–2056. doi: 10.1093/cercor/bhz221, PMID: 32037442

[ref42] Goldstein R. Z. Volkow N. D. (2011). Dysfunction of the prefrontal cortex in addiction: neuroimaging findings and clinical implications. Nat. Rev. Neurosci. 12, 652–669. doi: 10.1038/nrn3119, PMID: 22011681 PMC3462342

[ref43] Gosdin L. K. (2022). Alcohol consumption and binge drinking during pregnancy among adults aged 18–49 years—United States, 2018–2020. MMWR Morb. Mortal Wkly. Rep. 71, 10–13. doi: 10.15585/mmwr.mm7101a2, PMID: 34990444 PMC8735564

[ref44] Gosselin J. Gahagan S. Amiel-Tison C. (2005). The Amiel-Tison neurological assessment at term: conceptual and methodological continuity in the course of follow-up. Ment. Retard. Dev. Disabil. Res. Rev. 11, 34–51. doi: 10.1002/mrdd.20049, PMID: 15856442

[ref45] Grewen K. Burchinal M. Vachet C. Gouttard S. Gilmore J. H. Lin W.. (2014). Prenatal cocaine effects on brain structure in early infancy. NeuroImage 101, 114–123. doi: 10.1016/j.neuroimage.2014.06.070, PMID: 24999039 PMC4224027

[ref46] Grewen K. Salzwedel A. P. Gao W. (2015). Functional connectivity disruption in neonates with prenatal marijuana exposure. Front. Hum. Neurosci. 9:601. doi: 10.3389/fnhum.2015.00601, PMID: 26582983 PMC4631947

[ref47] Groeschel S. Vollmer B. King M. D. Connelly A. (2010). Developmental changes in cerebral grey and white matter volume from infancy to adulthood. Int. J. Dev. Neurosci. 28, 481–489. doi: 10.1016/j.ijdevneu.2010.06.004, PMID: 20600789

[ref48] Guerri C. (2002). Mechanisms involved in central nervous system dysfunctions induced by prenatal ethanol exposure. Neurotox. Res. 4, 327–335. doi: 10.1080/1029842021000010884, PMID: 12829422

[ref49] Harsan L. A. Poulet P. Guignard B. Steibel J. Parizel N. Loureiro de Sousa P.. (2006). Brain dysmyelination and recovery assessment by noninvasive in vivo diffusion tensor magnetic resonance imaging. J. Neurosci. Res. 83, 392–402. doi: 10.1002/jnr.20742, PMID: 16397901

[ref50] Harst L. Deckert S. Haarig F. Reichert J. Dinger J. Hellmund P.. (2021). Prenatal methamphetamine exposure: effects on child development. Dtsch. Arztebl. Int. 118, 313–319. doi: 10.3238/arztebl.m2021.0128, PMID: 34140080 PMC8295533

[ref51] Hauser K. F. Khurdayan V. K. Goody R. J. Nath A. Saria A. Pauly J. R. (2003). Selective vulnerability of cerebellar granule neuroblasts and their progeny to drugs with abuse liability. Cerebellum 2, 184–195. doi: 10.1080/14734220310016132, PMID: 14509568 PMC4306667

[ref52] Heilig M. Egli M. (2006). Pharmacological treatment of alcohol dependence: target symptoms and target mechanisms. Pharmacol. Ther. 111, 855–876. doi: 10.1016/j.pharmthera.2006.02.001, PMID: 16545872

[ref53] Hines M. (2010). Sex-related variation in human behavior and the brain. Trends Cogn. Sci. 14, 448–456. doi: 10.1016/j.tics.2010.07.005, PMID: 20724210 PMC2951011

[ref54] Hoff G. E. A.-J. Van Den Heuvel M. Benders M. J. N. L. Kersbergen K. J. de Vries L. S. (2013). On development of functional brain connectivity in the young brain. Front. Hum. Neurosci. 7:650. doi: 10.3389/fnhum.2013.00650 24115929 PMC3792361

[ref55] Hsiao S. H. West J. R. Mahoney J. C. Frye G. D. (1999). Postnatal ethanol exposure blunts upregulation of GABAA receptor currents in Purkinje neurons. Brain Res. 832, 124–135. doi: 10.1016/s0006-8993(99)01480-8, PMID: 10375658

[ref56] Huang A. S. Mitchell J. A. Haber S. N. Alia-Klein N. Goldstein R. Z. (2018). The thalamus in drug addiction: from rodents to humans. Philos. Trans. R. Soc. B Biol. Sci. 373:20170028. doi: 10.1098/rstb.2017.0028, PMID: 29352027 PMC5790826

[ref57] Irner T. B. (2012). Substance exposure in utero and developmental consequences in adolescence: a systematic review. Child Neuropsychol. 18, 521–549. doi: 10.1080/09297049.2011.628309, PMID: 22114955

[ref58] Jacobson S. W. Jacobson J. L. Molteno C. D. Warton C. M. R. Wintermark P. Hoyme H. E.. (2017). Heavy prenatal alcohol exposure is related to smaller corpus callosum in newborn MRI scans. Alcohol. Clin. Exp. Res. 41:965. doi: 10.1111/acer.13363 28247416 PMC5404976

[ref59] Jarlenski M. Krans E. E. (2021). Co-occurring substance use disorders identified among delivery hospitalizations in the United States. J. Addict. Med. 15, 504–507. doi: 10.1097/ADM.0000000000000792, PMID: 33273252 PMC8166954

[ref60] Ji X. Liu S. Li S. Li X. Luo A. Zhang X.. (2024). GABA in early brain development: a dual role review. Int. J. Dev. Neurosci. 84, 843–856. doi: 10.1002/jdn.10387, PMID: 39503050

[ref61] Jiang W. Merhar S. L. Zeng Z. Zhu Z. Yin W. Zhou Z.. (2022). Neural alterations in opioid-exposed infants revealed by edge-centric brain functional networks. Brain Commun. 4:112. doi: 10.1093/braincomms/fcac112, PMID: 35602654 PMC9117006

[ref62] Kaczkurkin A. N. Raznahan A. Satterthwaite T. D. (2019). Sex differences in the developing brain: insights from multimodal neuroimaging. Neuropsychopharmacology 44, 71–85. doi: 10.1038/s41386-018-0111-z, PMID: 29930385 PMC6235840

[ref63] Kidokoro H. Neil J. J. Inder T. E. (2013). New MR imaging assessment tool to define brain abnormalities in very preterm infants at term. Am. J. Neuroradiol. 34, 2208–2214. doi: 10.3174/ajnr.A3521, PMID: 23620070 PMC4163698

[ref64] Konczak J. Timmann D. (2007). The effect of damage to the cerebellum on sensorimotor and cognitive function in children and adolescents. Neurosci. Biobehav. Rev. 31, 1101–1113. doi: 10.1016/j.neubiorev.2007.04.014, PMID: 17599406

[ref65] Koob G. F. (1992). Drugs of abuse: anatomy, pharmacology and function of reward pathways. Trends Pharmacol. Sci. 13, 177–184. doi: 10.1016/0165-6147(92)90060-J, PMID: 1604710

[ref66] Kuhn B. N. Kalivas P. W. Bobadilla A.-C. (2019). Understanding addiction using animal models. Front. Behav. Neurosci. 13:262. doi: 10.3389/fnbeh.2019.00262, PMID: 31849622 PMC6895146

[ref67] Lambert B. L. Bauer C. R. (2012). Developmental and behavioral consequences of prenatal cocaine exposure: a review. J. Perinatol. 32, 819–828. doi: 10.1038/jp.2012.90, PMID: 22791278 PMC4143247

[ref68] Lean R. E. Smyser C. D. Brady R. G. Triplett R. L. Kaplan S. Kenley J. K.. (2022). Prenatal exposure to maternal social disadvantage and psychosocial stress and neonatal white matter connectivity at birth. Proc. Natl. Acad. Sci. USA 119, 1–8. doi: 10.1073/pnas.2204135119, PMID: 36219693 PMC9586270

[ref69] Lebel C. Deoni S. (2018). The development of brain white matter microstructure. NeuroImage 182, 207–218. doi: 10.1016/j.neuroimage.2017.12.097, PMID: 29305910 PMC6030512

[ref70] Lebel C. Treit S. Beaulieu C. (2019). A review of diffusion MRI of typical white matter development from early childhood to young adulthood. NMR Biomed. 32:e3778. doi: 10.1002/nbm.3778, PMID: 28886240

[ref71] Lind A. Parkkola R. Lehtonen L. Munck P. Maunu J. Lapinleimu H.. (2011). Associations between regional brain volumes at term-equivalent age and development at 2 years of age in preterm children. Pediatr. Radiol. 41, 953–961. doi: 10.1007/s00247-011-2071-x, PMID: 21534004

[ref72] Little B. Sud N. Nobile Z. Bhattacharya D. (2021). Teratogenic effects of maternal drug abuse on developing brain and underlying neurotransmitter mechanisms. Neurotoxicology 86, 172–179. doi: 10.1016/j.neuro.2021.08.007, PMID: 34391795

[ref73] Liu J. Grewen K. Gao W. (2022). Evidence for the normalization effects of medication for opioid use disorder on functional connectivity in neonates with prenatal opioid exposure. J. Neurosci. 42, 4555–4566. doi: 10.1523/JNEUROSCI.2232-21.2022, PMID: 35552232 PMC9172285

[ref74] Lovinger D. M. Alvarez V. A. (2017). Alcohol and basal ganglia circuitry: animal models. Neuropharmacology 122, 46–55. doi: 10.1016/j.neuropharm.2017.03.023, PMID: 28341206 PMC5479739

[ref75] Luján R. Shigemoto R. López-Bendito G. (2005). Glutamate and GABA receptor signalling in the developing brain. Neuroscience 130, 567–580. doi: 10.1016/j.neuroscience.2004.09.042, PMID: 15590141

[ref76] Lyu W. Thung K.-H. Huynh K. M. Wang L. Lin W. Ahmad S.. (2025). Functional development of the human cerebellum from birth to age five. Nat. Commun. 16:6350. doi: 10.1038/s41467-025-61465-y, PMID: 40640148 PMC12246265

[ref77] Mahabee-Gittens E. M. Kline-Fath B. M. Harun N. Folger A. T. He L. Parikh N. A. (2023). Prenatal tobacco smoke exposure and risk of brain abnormalities on magnetic resonance imaging at term in infants born very preterm. Am. J. Obstetr. Gynecol. MFM 5:100856. doi: 10.1016/j.ajogmf.2022.100856, PMID: 36592820 PMC9974884

[ref78] Matthews L. G. Walsh B. H. Knutsen C. Neil J. J. Smyser C. D. Rogers C. E.. (2018). Brain growth in the NICU: critical periods of tissue-specific expansion. Pediatr. Res. 83, 976–981. doi: 10.1038/pr.2018.4, PMID: 29320484 PMC6054136

[ref79] Merhar S. L. Kline J. E. Braimah A. Kline-Fath B. M. Tkach J. A. Altaye M.. (2021). Prenatal opioid exposure is associated with smaller brain volumes in multiple regions. Pediatr. Res. 90, 397–402. doi: 10.1038/s41390-020-01265-w, PMID: 33177677 PMC8110593

[ref80] Miller M. B. Picciotto M. R. (2016). “Nicotine pharmacology, abuse, and addiction” in Neuroscience in the 21st century. eds. Pfaff D. W. Volkow N. D. Rubenstein J. L., (New York, NY: Springer), 3659–3677.

[ref81] Monnelly V. J. Anblagan D. Quigley A. Cabez M. B. Cooper E. S. Mactier H.. (2018). Prenatal methadone exposure is associated with altered neonatal brain development. NeuroImage Clinic. 18, 9–14. doi: 10.1016/j.nicl.2017.12.033, PMID: 29326869 PMC5760461

[ref82] Morie K. P. Crowley M. J. Mayes L. C. Potenza M. N. (2019). Prenatal drug exposure from infancy through emerging adulthood: results from neuroimaging. Drug Alcohol Depend. 198, 39–53. doi: 10.1016/j.drugalcdep.2019.01.032, PMID: 30878766 PMC6688747

[ref83] Motz M. Motz M. Espinet S. D. Jeong J. J. Major D. Racine N.. (2011). The role of the mother-child relationship in developmental outcomes of infants and young children with and without prenatal alcohol exposure. Canad. J. Clinic. Pharmacol. 18, e544–e563.

[ref84] Mravčík V. Nechanská B. Gabrhelík R. Handal M. Mahic M. Skurtveit S. (2020). Socioeconomic characteristics of women with substance use disorder during pregnancy and neonatal outcomes in their newborns: a national registry study from the Czech Republic. Drug Alcohol Depend. 209:107933. doi: 10.1016/j.drugalcdep.2020.107933, PMID: 32109712

[ref85] Narkowicz S. Płotka J. Polkowska Ż. Biziuk M. Namieśnik J. (2013). Prenatal exposure to substance of abuse: a worldwide problem. Environ. Int. 54, 141–163. doi: 10.1016/j.envint.2013.01.011, PMID: 23454110

[ref86] Nishimaru H. Kakizaki M. (2009). The role of inhibitory neurotransmission in locomotor circuits of the developing mammalian spinal cord. Acta Physiol. 197, 83–97. doi: 10.1111/j.1748-1716.2009.02020.x, PMID: 19673737

[ref87] Nossin-Manor R. Card D. Morris D. Noormohamed S. Shroff M. M. Whyte H. E.. (2013). Quantitative MRI in the very preterm brain: assessing tissue organization and myelination using magnetization transfer, diffusion tensor and T1 imaging. NeuroImage 64, 505–516. doi: 10.1016/j.neuroimage.2012.08.086, PMID: 22982360

[ref88] Nygaard E. Slinning K. Moe V. Walhovd K. B. (2017). Cognitive function of youths born to mothers with opioid and poly-substance abuse problems during pregnancy. Child Neuropsychol. 23, 159–187. doi: 10.1080/09297049.2015.1092509, PMID: 26471942

[ref89] Oei J. L. (2018). Adult consequences of prenatal drug exposure. Intern. Med. J. 48, 25–31. doi: 10.1111/imj.13658, PMID: 29314518

[ref90] Ojeda J. Ávila A. (2019). Early actions of neurotransmitters during cortex development and maturation of reprogrammed neurons. Front. Synaptic Neurosci. 11:33. doi: 10.3389/fnsyn.2019.00033, PMID: 31824293 PMC6881277

[ref91] Ortigosa S. Friguls B. Joya X. Martinez S. Mariñoso M. L. Alameda F.. (2012). Feto-placental morphological effects of prenatal exposure to drugs of abuse. Reprod. Toxicol. 34, 73–79. doi: 10.1016/j.reprotox.2012.04.002, PMID: 22525318

[ref92] Ortinau C. Neil J. (2015). The neuroanatomy of prematurity: Normal brain development and the impact of preterm birth. Clin. Anat. 28, 168–183. doi: 10.1002/ca.22430, PMID: 25043926

[ref93] Ouyang M. Dubois J. Yu Q. Mukherjee P. Huang H. (2019). Delineation of early brain development from fetuses to infants with diffusion MRI and beyond. NeuroImage 185, 836–850. doi: 10.1016/j.neuroimage.2018.04.017, PMID: 29655938 PMC6185831

[ref94] Pando-Naude V. Toxto S. Fernandez-Lozano S. Parsons C. E. Alcauter S. Garza-Villarreal E. A. (2021). Gray and white matter morphology in substance use disorders: a neuroimaging systematic review and meta-analysis. Transl. Psychiatry 11, 1–18. doi: 10.1038/s41398-020-01128-2 33431833 PMC7801701

[ref95] Peterson B. S. Anderson A. W. Ehrenkranz R. Staib L. H. Tageldin M. Colson E.. (2003). Regional brain volumes and their later neurodevelopmental correlates in term and preterm infants. Pediatrics 111, 939–948. doi: 10.1542/peds.111.5.939, PMID: 12728069

[ref96] Popova S. Lange S. Probst C. Gmel G. Rehm J. (2017). Estimation of national, regional, and global prevalence of alcohol use during pregnancy and fetal alcohol syndrome: a systematic review and meta-analysis. Lancet Glob. Health 5, e290–e299. doi: 10.1016/S2214-109X(17)30021-9, PMID: 28089487

[ref97] Pulli E. P. Kumpulainen V. Kasurinen J. H. Korja R. Merisaari H. Karlsson L.. (2018). Prenatal exposures and infant brain: review of magnetic resonance imaging studies and a population description analysis. Hum. Brain Mapp. 40, 1987–2000. doi: 10.1002/hbm.24480, PMID: 30451332 PMC6865387

[ref98] Qiu D. Tan L.-H. Zhou K. Khong P.-L. (2008). Diffusion tensor imaging of normal white matter maturation from late childhood to young adulthood: voxel-wise evaluation of mean diffusivity, fractional anisotropy, radial and axial diffusivities, and correlation with reading development. NeuroImage 41, 223–232. doi: 10.1016/j.neuroimage.2008.02.023, PMID: 18395471

[ref99] Radhakrishnan R. Elsaid N. M. H. Sadhasivam S. Reher T. A. Hines A. C. Yoder K. K.. (2021). Resting state functional MRI in infants with prenatal opioid exposure—a pilot study. Neuroradiology 63, 585–591. 32978671 10.1007/s00234-020-02552-3 PMC9162800

[ref100] Raschle N. Zuk J. Ortiz-Mantilla S. Sliva D. D. Franceschi A. Grant P. E.. (2012). Pediatric neuroimaging in early childhood and infancy: challenges and practical guidelines. Ann. N. Y. Acad. Sci. 1252, 43–50. doi: 10.1111/j.1749-6632.2012.06457.x, PMID: 22524338 PMC3499030

[ref101] Rees S. Harding R. (2004). Brain development during fetal life: influences of the intra-uterine environment. Neurosci. Lett. 361, 111–114. doi: 10.1016/j.neulet.2004.02.002, PMID: 15135906

[ref102] Riley E. P. Infante M. A. Warren K. R. (2011). Fetal alcohol Spectrum disorders: an overview. Neuropsychol. Rev. 21, 73–80. doi: 10.1007/s11065-011-9166-x, PMID: 21499711 PMC3779274

[ref103] Roos A. Fouche J.-P. Ipser J. C. Narr K. L. Woods R. P. Zar H. J.. (2021). Structural and functional brain network alterations in prenatal alcohol exposed neonates. Brain Imaging Behav. 15, 689–699. doi: 10.1007/s11682-020-00277-8, PMID: 32306280 PMC7572489

[ref104] Ross E. J. Graham D. L. Money K. M. Stanwood G. D. (2015). Developmental consequences of fetal exposure to drugs: what we know and what we still must learn. Neuropsychopharmacology 40, 61–87. doi: 10.1038/npp.2014.147, PMID: 24938210 PMC4262892

[ref105] Roy T. S. Seidler F. J. Slotkin T. A. (2002). Prenatal nicotine exposure evokes alterations of cell structure in Hippocampus and somatosensory cortex. J. Pharmacol. Exp. Ther. 300, 124–133. doi: 10.1124/jpet.300.1.124, PMID: 11752107

[ref106] Salisbury A. L. Ponder K. L. Padbury J. F. Lester B. M. (2009). Fetal effects of psychoactive drugs. Clin. Perinatol. 36, 595–619. doi: 10.1016/j.clp.2009.06.002, PMID: 19732616 PMC2767264

[ref107] Salmanzadeh H. Ahmadi-Soleimani S. M. Pachenari N. Azadi M. Halliwell R. F. Rubino T.. (2020). Adolescent drug exposure: a review of evidence for the development of persistent changes in brain function. Brain Res. Bull. 156, 105–117. doi: 10.1016/j.brainresbull.2020.01.007, PMID: 31926303

[ref108] Salzwedel A. Chen G. Chen Y. Grewen K. Gao W. (2020). Functional dissection of prenatal drug effects on baby brain and behavioral development. Hum. Brain Mapp. 41, 4789–4803. doi: 10.1002/hbm.25158, PMID: 32779835 PMC7643353

[ref109] Salzwedel A. P. Grewen K. M. Goldman B. D. Gao W. (2016). Thalamocortical functional connectivity and behavioral disruptions in neonates with prenatal cocaine exposure. Neurotoxicol. Teratol. 56, 16–25. doi: 10.1016/j.ntt.2016.05.009, PMID: 27242332 PMC4935611

[ref110] Salzwedel A. P. Grewen K. M. Vachet C. Gerig G. Lin W. Gao W. (2015). Prenatal drug exposure affects neonatal brain functional connectivity. J. Neurosci. 35, 5860–5869. doi: 10.1523/JNEUROSCI.4333-14.2015, PMID: 25855194 PMC4388938

[ref111] SAMHSA (2020). Substance Abuse and Mental Health Services Administration: Key substance use and mental health indicators in the United States: Results from the 2019 National Survey on drug use and health. (no. HHS publication no PEP20-07-01-001; NSDUH series H-55). Rockville, MD: Center for Behavioral Health Statistics and Quality.

[ref112] Sanjari Moghaddam H. Mobarak Abadi M. Dolatshahi M. Bayani Ershadi S. Abbasi-Feijani F. Rezaei S.. (2021). Effects of prenatal methamphetamine exposure on the developing human brain: a systematic review of neuroimaging studies. ACS Chem. Neurosci. 12, 2729–2748. doi: 10.1021/acschemneuro.1c00213, PMID: 34297546 PMC8763371

[ref113] Sesack S. R. Grace A. A. (2010). Cortico-basal ganglia reward network: microcircuitry. Neuropsychopharmacology 35, 27–47. doi: 10.1038/npp.2009.93, PMID: 19675534 PMC2879005

[ref114] Sirnes E. Griffiths S. T. Aukland S. M. Eide G. E. Elgen I. B. Gundersen H. (2018). Functional MRI in prenatally opioid-exposed children during a working memory-selective attention task. Neurotoxicol. Teratol. 66, 46–54. doi: 10.1016/j.ntt.2018.01.010, PMID: 29408607

[ref115] Sled J. G. Nossin-Manor R. (2013). Quantitative MRI for studying neonatal brain development. Neuroradiology 55, 97–104. doi: 10.1007/s00234-013-1235-9 23872867

[ref116] Smith A. M. Mioduszewski O. Hatchard T. Byron-Alhassan A. Fall C. Fried P. A. (2016). Prenatal marijuana exposure impacts executive functioning into young adulthood: an fMRI study. Neurotoxicol. Teratol. 58, 53–59. doi: 10.1016/j.ntt.2016.05.010, PMID: 27263090

[ref117] Song S.-K. Sun S.-W. Ramsbottom M. J. Chang C. Russell J. Cross A. H. (2002). Dysmyelination revealed through MRI as increased radial (but unchanged axial) diffusion of water. NeuroImage 17, 1429–1436. doi: 10.1006/nimg.2002.1267, PMID: 12414282

[ref118] Squeglia L. M. Gray K. M. (2016). Alcohol and drug use and the developing brain. Curr. Psychiatry Rep. 18:46. doi: 10.1007/s11920-016-0689-y, PMID: 26984684 PMC4883014

[ref119] Squeglia L. M. Jacobus J. Tapert S. F. (2009). The influence of substance use on adolescent brain development. Clin. EEG Neurosci. 40, 31–38. doi: 10.1177/155005940904000110, PMID: 19278130 PMC2827693

[ref120] Steven A. J. Zhuo J. Melhem E. R. (2014). Diffusion kurtosis imaging: an emerging technique for evaluating the microstructural environment of the brain. Am. J. Roentgenol. 202, W26–W33. doi: 10.2214/AJR.13.11365, PMID: 24370162

[ref121] Stiles J. Jernigan T. L. (2010). The basics of brain development. Neuropsychol. Rev. 20, 327–348. doi: 10.1007/s11065-010-9148-4, PMID: 21042938 PMC2989000

[ref122] Takahashi K. A. Linden D. J. (2000). Cannabinoid receptor modulation of synapses received by cerebellar Purkinje cells. J. Neurophysiol. 83, 1167–1180. doi: 10.1152/jn.2000.83.3.1167, PMID: 10712447

[ref123] Tavella R. A. De Abreu V. O. M. Muccillo-Baisch A. L. Da Silva Júnior F. M. R. (2020). Prevalence of illicit drug use during pregnancy: a global perspective. An. Acad. Bras. Cienc. 92:e20200302. doi: 10.1590/0001-3765202020200302, PMID: 33295578

[ref124] Taylor P. A. Jacobson S. W. van der Kouwe A. Molteno C. D. Chen G. Wintermark P.. (2015). A DTI-based tractography study of effects on brain structure associated with prenatal alcohol exposure in newborns. Hum. Brain Mapp. 36, 170–186. doi: 10.1002/hbm.22620, PMID: 25182535 PMC4311768

[ref125] Terasaki L. S. Gomez J. Schwarz J. M. (2016). An examination of sex differences in the effects of early-life opiate and alcohol exposure. Philos. Trans. R. Soc. B Biol. Sci. 371:20150123. doi: 10.1098/rstb.2015.0123, PMID: 26833841 PMC4785906

[ref126] Thompson V. B. Heiman J. Chambers J. B. Benoit S. C. Buesing W. R. Norman M. K.. (2009). Long-term behavioral consequences of prenatal MDMA exposure. Physiol. Behav. 96, 593–601. doi: 10.1016/j.physbeh.2008.12.013, PMID: 19162054 PMC2649789

[ref127] Tierney A. L. Nelson C. A. (2009). Brain development and the role of experience in the early years. Zero to Three 30, 9–13. 23894221 PMC3722610

[ref128] Tomášková A. Šlamberová R. Černá M. (2020). Influence of prenatal methamphetamine abuse on the brain. Epigenomes 4:14. doi: 10.3390/epigenomes4030014, PMID: 34968287 PMC8594709

[ref129] Townsel C. Metz T. D. Bunik M. (2021). The term newborn: prenatal substance exposure. Clin. Perinatol. 48, 631–646. doi: 10.1016/j.clp.2021.05.011, PMID: 34353584

[ref130] Umer A. Watson E. Lilly C. Woods S. Lefeber C. Breyel J.. (2023). Substance exposure and adverse neonatal outcomes: a population-based cohort study. J. Pediatr. 256, 70–76. doi: 10.1016/j.jpeds.2022.11.040, PMID: 36513212

[ref131] US Department of Health and Human Services. (2016). The neurobiology of substance use, misuse, and addiction. In Facing addiction in America: The surgeon general’s report on alcohol, drugs, and health [internet]. US Department of Health and Human Services. Available online at: https://www.ncbi.nlm.nih.gov/books/NBK424849/ (Accessed January 26, 2025).

[ref132] Vishnubhotla R. V. Ahmad S. T. Zhao Y. Radhakrishnan R. (2024). Impact of prenatal marijuana exposure on adolescent brain structural and functional connectivity and behavioural outcomes. Brain Commun. 6:fcae001. doi: 10.1093/braincomms/fcae001, PMID: 38444906 PMC10914455

[ref133] Walhovd K. B. Watts R. Amlien I. Woodward L. J. (2012). Neural tract development of infants born to methadone-maintained mothers. Pediatr. Neurol. 47, 1–6. doi: 10.1016/j.pediatrneurol.2012.04.008, PMID: 22704008

[ref134] Wang S. (2019). Historical review: opiate addiction and opioid receptors. Cell Transplant. 28, 233–238. doi: 10.1177/0963689718811060, PMID: 30419763 PMC6425114

[ref135] Warton F. L. Meintjes E. M. Warton C. M. R. Molteno C. D. Lindinger N. M. Carter R. C.. (2018a). Prenatal methamphetamine exposure is associated with reduced subcortical volumes in neonates. Neurotoxicol. Teratol. 65, 51–59. APA PsycInfo. Doi:10.1016/j.ntt.2017.10.005. doi: 10.1016/j.ntt.2017.10.005, PMID: 29069607 PMC5803390

[ref136] Warton F. L. Molteno C. D. Warton C. M. R. Wintermark P. Lindinger N. M. Dodge N. C.. (2021). Maternal choline supplementation mitigates alcohol exposure effects on neonatal brain volumes. Alcohol. Clin. Exp. Res. 45, 1762–1774. APA PsycInfo. Doi:10.1111/acer.14672. doi: 10.1111/acer.14672, PMID: 34342017 PMC8526390

[ref137] Warton F. L. Taylor P. A. Warton C. M. R. Molteno C. D. Wintermark P. Lindinger N. M.. (2018b). Prenatal methamphetamine exposure is associated with corticostriatal white matter changes in neonates. Metab. Brain Dis. 33, 507–522 Academic Search Premier. doi: 10.1007/s11011-017-0135-9, PMID: 29063448 PMC5866741

[ref138] Warton F. L. Taylor P. A. Warton C. M. R. Molteno C. D. Wintermark P. Zöllei L.. (2020). Reduced fractional anisotropy in projection, association, and commissural fiber networks in neonates with prenatal methamphetamine exposure. Dev. Neurobiol. 80, 381–398. doi: 10.1002/dneu.22784, PMID: 33010114 PMC7855045

[ref139] Willford J. A. Chandler L. S. Goldschmidt L. Day N. L. (2010). Effects of prenatal tobacco, alcohol and marijuana exposure on processing speed, visual–motor coordination, and interhemispheric transfer. Neurotoxicol. Teratol. 32, 580–588. doi: 10.1016/j.ntt.2010.06.004, PMID: 20600845 PMC2975798

[ref140] Willford J. Smith C. Kuhn T. Weber B. Richardson G. Willford J.. (2016). “Review of current neuroimaging studies of the effects of prenatal drug exposure: brain structure and function” in Recent advances in drug addiction research and clinical applications. eds. Meil W. M. Ruby C. L., (London, UK: IntechOpen). doi: 10.5772/63389

[ref141] Winklewski P. J. Sabisz A. Naumczyk P. Jodzio K. Szurowska E. Szarmach A. (2018). Understanding the physiopathology behind axial and radial diffusivity changes—what do we know? Front. Neurol. 9:92. doi: 10.3389/fneur.2018.00092, PMID: 29535676 PMC5835085

[ref142] Xu X. Sun C. Sun J. Shi W. Shen Y. Zhao R.. (2022). Spatiotemporal atlas of the fetal brain depicts cortical developmental gradient. J. Neurosci. 42, 9435–9449. doi: 10.1523/JNEUROSCI.1285-22.2022, PMID: 36323525 PMC9794379

[ref143] Young-Wolff K. C. Slama N. E. Sarovar V. Terplan M. Ansley D. Adams S. R.. (2022). Trends in self-reported and biochemically verified cocaine and methamphetamine use among pregnant individuals in northern California, 2011-2019. JAMA Netw. Open 5:e2248055. doi: 10.1001/jamanetworkopen.2022.48055, PMID: 36542384 PMC9857285

[ref144] Yuan Q. Rubic M. Seah J. Rae C. Wright I. M. R. Kaltenbach K.. (2014). Do maternal opioids reduce neonatal regional brain volumes? A pilot study. J. Perinatol. 34, 909–913. doi: 10.1038/jp.2014.111, PMID: 24945162

[ref145] Zhang H. Schneider T. Wheeler-Kingshott C. A. Alexander D. C. (2012). NODDI: practical in vivo neurite orientation dispersion and density imaging of the human brain. NeuroImage 61, 1000–1016. doi: 10.1016/j.neuroimage.2012.03.072, PMID: 22484410

