# Correction: Prenatal substance exposure and infant neurodevelopment: a review of magnetic resonance imaging studies

**DOI:** 10.3389/fnhum.2025.1717377

**Published:** 2025-10-16

**Authors:** Leela Shah, Christy D. Yoon, Alessandra M. LaJeunesse, Lilly G. Schirmer, Emma W. Rapallini, Elizabeth M. Planalp, Douglas C. Dean

**Affiliations:** ^1^Department of Neuroscience Training Program, University of Wisconsin-Madison, Madison, WI, United States; ^2^Waisman Center, University of Wisconsin-Madison, Madison, WI, United States; ^3^Department of Pediatrics, University of Wisconsin-Madison, Madison, WI, United States; ^4^Department of Medical Physics, University of Wisconsin-Madison, Madison, WI, United States

**Keywords:** prenatal substance exposure, magnetic resonance imaging, infant brain development, diffusion MRI, structural MRI, functional MRI, developmental outcomes

There was a mistake in the caption of Figure 1 as published. The Figure contains the incorrect citation to Biorender. The corrected caption of Figure 1 appears below.

Figure 1. Summary of imaging findings across substance exposures highlighting target regions. Aspects of this image created in BioRender. Shah, L. (2025) https://BioRender.com/tukao8z.

The original version of this article has been updated.

